# How do tumor-associated neutrophils regulate the microenvironmental landscape of brain tumors: Delivery of nano-particles through BBB

**DOI:** 10.1371/journal.pcbi.1013906

**Published:** 2026-01-23

**Authors:** Haneol Cho, Junho Lee, Sean Lawler, Yangjin Kim

**Affiliations:** 1 Department of Mathematics, Konkuk University, Seoul, Republic of Korea; 2 Natural Product Informatics Research Center, Gangneung Natural Products Institute, Korea Institute of Science and Technology, Gangneung, Republic of Korea; 3 Department of Pathology and Laboratory Medicine, Legorreta Brown Cancer Center, Brown University, Providence, Rhode Island, United States of America; University of Southern California, UNITED STATES OF AMERICA

## Abstract

Glioblastoma multiforme (GBM) is the most aggressive form of brain cancer with the very poor survival and high recurrence rate. Tumor-associated neutrophils (TANs) play a pivotal role in regulation of the tumor microenvironment. In this study, we developed a new mathematical model of the critical GBM-TAN interaction in the heterogeneous brain tissue. The model reveals that the dual and complex role of TANs (either anti-tumorigenic N1 and the pro-tumorigenic N2 type) regulates the phenotypic trajectory of the evolution of tumor growth and the invasive patterns in white and gray matter via mediators such as IFN-*β* and TGF-*β*. We investigated the effect of normalizing the immune environment on glioma growth by applying a therapeutic antibody and developed several strategies for eradication of tumor cells by neutrophil-mediated transport of nanoparticles. We also developed a strategy of combination therapy (surgery + Trojan neutrophils) for effective control of the infiltration of the glioma cells in one hemisphere before crossing the corpus callosum (CC) in order to prevent recurrence in the other hemisphere. This alternative approach compared to the extended resection of the glioma including CC or butterfly GBM may provide the greater anti-tumor efficacy and reduce side effects such as cognitive impairment.

## Author summary

Glioblastoma (GBM), the most aggressive form of brain cancer, is characterized by rapid growth and aggressive invasion even after surgery, leading to very poor survival. While neutrophils circulating in the blood are an important part of the immune system as the first line of defense against cancer, tumor-associated neutrophils (TANs) play a dual role in regulation of the tumor microenvironment. We used mathematical models to investigate the critical GBM-TAN interaction in the heterogeneous brain tissue. The model showed the complex phenotypic switches between N1 and N2 phenotypes in the evolution of tumor growth and the invasive patterns under biophysical conditions in white and gray matter via regulation of biochemical mediators. In this study, we developed several strategies for killing GBM cells by a therapeutic antibody against N2 TANs and neutrophil-mediated transport of anti-cancer nanoparticles. We also illustrated how a combination therapy (surgery + Trojan neutrophils) effectively control glioma infiltration through the corpus callosum, preventing regrowth of GBM in the contralateral hemisphere. This new approach may provide greater anti-tumor efficacy and reduce side effects such as cognitive impairment as a result of damage to normal brain tissue.

## Introduction

The brain glioblastoma (GBM), one of deadliest cancers in human [1–4], is characterized by fast growth and aggressive infiltration [5], typically leading to the high level of local diffusive invasion and poor clinical outcomes [6,7]. Several studies have shown that these invasive tumor cells tend to migrate along the blood vessels (BVs) and myelinated axons, a white matter tract located in a corpus callosum (CC) which connects the two brain hemispheres [8–10]. BVs in the brain are characterized by tight junctions and have selective, nonlinear permeability, hindering the delivery of anti-cancer drugs, forming the blood–brain barrier (BBB) [11,12]. Heterogeneity in human brain tissues, consisting of white and grey matter, makes drug transport even unpredictable due to different diffusion properties [13,14]. These factors contribute to the severe challenges in eradicating the GBM cells [1,15].

Neutrophils, the most abundant circulating leukocytes, are known as the first line of defense against bacterial and fungal infections [16]. However, until recently their critical role in tumor growth and other processes in cancer progression was poorly understood due to the short half-life of most of populations from the bone marrow and other technical factors [17]. The tumor microenvironment (TME) consists of blood vessels, extracellular matrix (ECM), cytokines and immune cells including macrophages [18], natural killer (NK) cells [19,20], and tumor-associated neutrophils (TANs) [21–23]. Despite the critical role of TANs in regulation of GBM-immune interactions and invasive capacity of glioma cells in TME, the detailed mechanisms involved are poorly understood [16]. Two distinct TAN phenotypes exist in a given TME: (i) anti-tumorigenic N1 TANs, (ii) pro-tumorigenic N2 TANs [24–26]. The critical transition from N1 to N2 TANs and reverse switch are mediated by transforming growth factor beta (TGF-*β*) [26–28] and interferon beta (IFN-*β*) [29–32], respectively. Thus, inhibition of N2 TANs can increase anti-tumor efficacy by blocking the anti-tumor effect of N2 TANs and normalizing the immunity of N1 TANs, leading to the development of anti-N2 TAN drugs such as the Ly6G antibody [33]. Recent work showed that neutrophils are recruited by tumor-induced macrophages and can physically communicate with tumor cells to increase proliferative and invasive capacity, leading to increased tumor aggressiveness with a signaling niche in breast cancer [34].

Surgical resection is a major first line of treatment for GBM in an effort to preserve neurological function [35,36], but does not lead to complete eradication of tumor cells, leaving infiltrating cancer cells and regrowth after aggressive invasion toward the other part of brain. A typical adjuvant therapy, chemotherapy, is not effective in killing cancer cells due to weak penetration of drugs from two major brain obstacles: BBB and blood-tumor barrier (BTB) [37–39]. TANs can be used as a drug delivery system for anticancer drugs by taking advantage of effortless transport of therapeutics through chemotaxis toward the tumor and their ability to cross the BBB and BTB [16]. Thus well-controlled anti-cancer strategies by these *Trojan horses* may empower eradication of cancerous cells in heterogenous brain after a macroscale tumor debulking by surgery [40]. For example, in a melanoma model, the delivery of CXCL1 hydrogels and anti-cancer nanoparticles with neutrophils as a carrier showed strong anti-tumor efficacy via the chemotactic movement and phagocytosis of neutrophils [40]. Nanoparticle-based delivery systems can be very effective when the chemokines are injected at the periphery of surgical resection site [40–42]. In recent studies, Ding [43] reported that semiconducting polymer nano-therapeutics (SPCFe/siP) can used for efficient delivery into the glioma tissue through the BBB via neutrophil-mediated transport in ferroptosis-immunotherapy.

Butterfly glioblastoma (BG) belongs to a subclass of glioblastoma with a symmetric, bihemispheric shape, arising from glioma cells that cross CC [44,45]. BG is often associated with a poor prognosis (median overall survival of 5.5 months) [45]. Surgical removal of a GBM mass near the CC leads to the active spread of remaining tumor cells with accelerated growth speed and growth in other hemisphere after crossing the CC [46–48], thus resulting in poor prognosis [49,50]. While maximal removal of the GMB mass near the CC is positively correlated with longer overall survival [51], it often causes various side effects including cognitive impairment [52,53].

Previous modeling studies illustrated that TANs contribute to the complex tumor-immune system in lung cancer development by using mathematical models in a simple form of ordinary differential equations (ODEs) [21,23]. However, the fundamental mechanism of the complex interplay of TANs with glioma cells in the spatial heterogenous brain (white and gray matter) in regulation of cellular infiltration, recurrence, and local metastasis of glioma is poorly understood.

Our mathematical model consists of

(i) a system of partial differential equations (PDEs) involving the following key variables at space x and time *t*:


$$N_{1}(x,t)=\text{density of N1 TANs},\;N_{2}(x,t)=\text{density of N2 TANs},\;A(x,t)=\text{concentration of N2 antibody},\;S(x,t)=\text{concentration of IFN-}\beta ,\;G(x,t)=\text{concentration of TGF-}\beta ,\;n(x,t)=\text{density of glioma cells}.$$

(ii) a hybrid multi-scale mathematical model involving cellular automata (CA) model [54,55] and reaction-diffusion equations of diffusible molecules.

**Fig 1:**
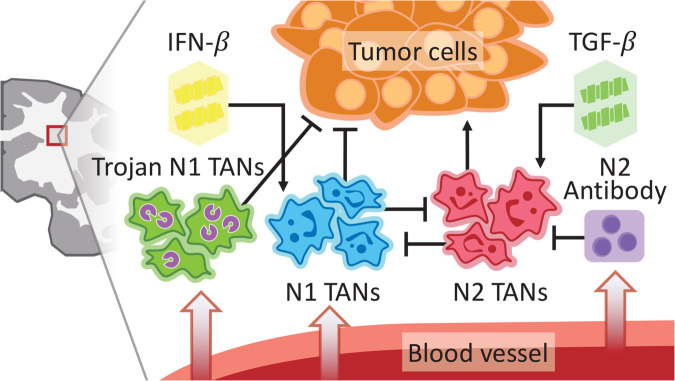
A conceptual illustration of TME including tumor cells, various TANs, and immune-related stimuli. Bar signs connecting two elements represent an effect of a given element toward the targeting element: arrows and hammerheads indicate promotion and inhibition, respectively.

These mathematical models are used (i) to investigate how N1/N2 TANs interact with glioma cells to regulate tumor growth in the heterogenous brain environment including white and gray matter, (ii) to investigate how this cross talk between neutrophils affects glioma cell infiltration, (iii) to design new strategies of Trojan N1 TANs to inhibit tumor growth by overcoming the BBB, (iv) to study the critical role of neutrophils in formation of butterfly glioma, and (v) to develop anti-invasion strategies to block recurrence of tumor population. Fig 1 depicts the GBM-immune interactions in the brain TME.

## Materials and methods

### Mathematical models

**Fig 2:**
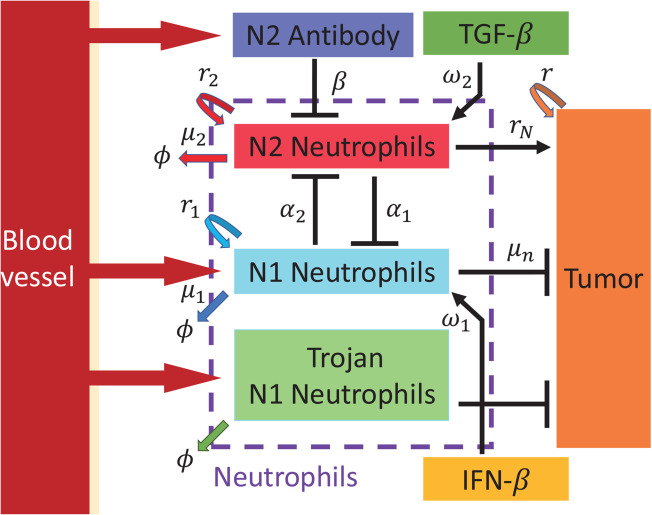
A schematic diagram describing the dynamics of TANs in the TME.

We developed a mathematical model of tumor-immune interactions in the brain TME and cell migration pattern in the presence of gray and white matter, a critical step in local spread [16,56] and recurrence of GBM, based on mutual interactions between N1/N2 TANs and tumor cells (Fig 2).

### Densities of N1 TANs ($=N_{1}(x,t)$) and N2 TANs ($=N_{2}(x,t)$)

We assume that (i) N1 and N2 TANs infiltrate the non-homogeneous brain tissue with space-dependent random-walk motility constant $D_{1}(x),D_{2}(x)$, respectively. We take much lower diffusion coefficients of N1 and N2 TANs (and other variables) in gray matter relative to ones in white matter when applied in later sections where we take into account geometry of white and gray matters in computational domain. (ii) N1 and N2 TANs grow at a rate $r_{1},r_{2}$, with the intrinsic carrying capacity *K*_1_, *K*_2_, respectively. (iii) It is well established that N1 TANs play the anti-tumorigenic role and N2 TANs have a pro-tumorigenic effect on tumor growth [24–26]. These mutual antagonism between N1 and N2 TANs has been well studied [23]. Herein, we adopted a Lotka-Volterra competition model with competition parameters $\alpha_{1},\alpha_{2}$ to illustrate the relationships of N1 TANs (*N*_1_) and N2 TANs (*N*_2_) [57] for competition between N1 and N2 TANs. The IFN-*β* (*S*) in the carrying capacity term enhance growth of N1 TANs with the promotion strength *w*_1_ while TGF-*β* (*G*) in the carrying capacity term promote growth of N2 TANs with the promotion strength *w*_2_ as illustrated in experimental studies [24,58]. (iv) N1 and N2 TANs are removed from the system at a rate $\mu_{1}$, $\mu_{2}$, respectively. (v) In the presence of antibody (*A*) against N2 TANs, N2 TANs are killed by the antibody at a rate *β*.

Then, the mass balance equations for the density of N1 TANs $N_{1}(x,t)$ and N2 TANs $N_{2}(x,t)$ under assumptions above are given by

$$\frac{\partial N_{1}}{\partial t}=\underset{\text{Invasion}}{\underbrace{\nabla \cdot (D_{1}(x)\nabla N_{1})}}+\underset{\text{Growth}}{\underbrace{r_{1}N_{1}\left(1-\frac{N_{1}}{w_{1}S+K_{1}}\right)}}-\underset{\text{Competition}}{\underbrace{\alpha_{1}N_{1}N_{2}}}-\underset{\text{Natural Decay}}{\underbrace{\mu_{1}N_{1}}},$$ (1)

$$\frac{\partial N_{2}}{\partial t}=\underset{\text{Invasion}}{\underbrace{\nabla \cdot (D_{2}(x)\nabla N_{2})}}+\underset{\text{Growth}}{\underbrace{r_{2}N_{2}\left(1-\frac{N_{2}}{\omega_{2}G+K_{2}}\right)}}-\underset{\text{Competition}}{\underbrace{\alpha_{2}N_{1}N_{2}}}\;\;\;-\underset{\text{Natural Decay}}{\underbrace{\mu_{2}N_{2}}}-\underset{\text{Killing by antibody}}{\underbrace{\beta N_{2}A}}.$$ (2)

### Concentration of antibody (=A(x,t))

In a similar fashion, by taking into account space-dependent diffusion, injection and decay of the antibody, we have the governing equation of N2 antibody (*A*):

$$\frac{\partial A}{\partial t}=\underset{\text{Diffusion}}{\underbrace{\nabla \cdot (D_{A}(x)\nabla A)}}+\underset{\text{Injection}}{\underbrace{\lambda_{A}(t)I_{\Omega_{A}}}}-\underset{\text{Decay}}{\underbrace{\mu_{A}A}}.$$ (3)

where $D_{A}(x)$ is the space-dependent diffusion coefficient of antibody, $\lambda_{A}(t)$ is the time-dependent injection rate of antibody over the sub-domain $\Omega_{A}$ ($\Omega_{A}\subset \Omega$), and $\mu_{A}$ represents the decay rate of the antibody. Here, the indicator function $I_{\Omega_{A}}$ is given by

$$I_{\Omega_{A}}(x)=\left\{ \begin{array}{c} 1\;\;\;\text{ if }x\in \Omega_{A}\\ 0\;\;\;\text{ otherwise. } \end{array}\right.$$ (4)

### Density of tumor cells (=n(x,t))

We assume that (i) tumor growth follows a nonlinear logistic growth at a basic rate *r*, with a carrying capacity *n*_0_. We also assume an additive growth enhancement from N2 TANs [23–26] with the Hill-type switching function $r_{N}\frac{{N_{2}}^{2}}{k^{2}+{N_{2}}^{2}}$ where *r*_*N*_ is the enhancement scaling parameter and *k* is the Hill-type coefficient . (ii) tumor cells diffuse in a heterogeneous brain tissue including white and gray matter in the brain tissue [56] with the space-dependent random motility constant $D_{n}(x)$, (iii) tumor cells are killed by N1 TANs at a rate $\mu_{n}$.

Under these assumptions, the governing equation for the tumor density is given by

$$\frac{\partial n}{\partial t}=\underset{\text{Movement}}{\underbrace{\nabla \cdot (D_{n}(x)\nabla n)}}+\underset{\text{Growth}}{\underbrace{r\left(1+r_{N}\frac{{N_{2}}^{2}}{k^{2}+{N_{2}}^{2}}\right)n\left(1-\frac{n}{n_{0}}\right)}}-\underset{\text{Cell killing}}{\underbrace{\mu_{n}N_{1}n}}.$$ (5)

### Concentration of IFN-*β* (=S(x,t))

In a similar fashion, we have the following form of the governing equations of IFN-*β* concentration:

$$\frac{\partial S}{\partial t}=\underset{\text{Diffusion}}{\underbrace{\nabla \cdot (D_{S}(x)\nabla S)}}+\underset{\text{Source}}{\underbrace{\lambda_{S}I_{S}}}-\underset{\text{Natural Decay}}{\underbrace{\mu_{S}S}}$$ (6)

where $D_{S}(x)$ is the space-dependent diffusion coefficient of IFN-*β*, $\lambda_{S}$ is the secretion rate of IFN-*β* and, $\mu_{S}$ is the decay rate of IFN-*β*. Here, *I*_*S*_ is an indicator function, giving 1 when injected and 0 otherwise.

### Concentration of TGF-*β* (=G(x,t))

Tumor cells secrete TGF-*β*, which mediates the critical transition from N1 to N2 TANs [24,58]. Thus, TGF-*β* inhibitors (for example, Galunisertib (LY2157299)) can be used to inhibit the N2 TAN-dominant TME [59–61]. Then, the governing equation of the TGF-*β* concentration is given by

$$\frac{\partial G}{\partial t}=\underset{\text{Diffusion}}{\underbrace{\nabla \cdot (D_{G}(x)\nabla G)}}+\underset{\text{Production}}{\underbrace{\lambda_{G}n}}-\underset{\text{Natural Decay}}{\underbrace{\mu_{G}G}}$$ (7)

where $D_{G}(x)$ is the space-dependent diffusion coefficient of TGF-*β*, $\lambda_{G}$ is the secretion rate of TGF-*β* from tumor cells and, $\mu_{G}$ is the decay rate of TGF-*β*.

### Boundary conditions and initial conditions

In the following simulations we prescribe Neumann boundary conditions on the boundary ∂Ω as follows:

$$J_{1}\cdot \nu =0,\;\;J_{2}\cdot \nu =0,\;\;J_{A}\cdot \nu =0,\;\;J_{n}\cdot \nu =0,$$ (8)

$$(D_{S}\nabla S)\cdot \nu =0,\;\;(D_{G}\nabla G)\cdot \nu =0,$$ (9)

where ν is the unit outer normal vector.

Finally, we prescribe initial conditions in Ω

$$N_{1}(x,0)=N_{1,0}(x),\;N_{2}(x,0)=N_{2,0}(x),\;A(x,0)=A_{0}(x),n(x,0)=n_{0}(x),\;S(x,0)=S_{0}(x),\;G(x,0)=G_{0}(x).$$ (10)

Parameter estimation is also performed in the S1 Text. Parameter values are given in Table 1. All the simulations for the PDE model (1)–(10) were performed using a finite difference method with central difference scheme and $h_{x}=h_{y}=0.01$ in 2D domain [0,1]^2^.

**Table 1:** Parameter set used in the model. *Est = Estimated.

Par	Description	Value	Ref
Production rates & carrying capacities of TANs & stimuli			
*r*_1_	Growth rate of N1 TANs	$8.0\times 10^{-2}$ *h*^−1^	Est
*r*_2_	Growth rate of N2 TANs	$8.0\times 10^{-2}$ *h*^−1^	Est
*K*_1_	Carrying capacity of N1 TANs	$5.0\times 10^{6}$ *cells*/*cm*^3^	Est
*K*_2_	Carrying capacity of N2 TANs	$4.0\times 10^{6}$ *cells*/*cm*^3^	Est
$\lambda_{A}$	Injection rate of N2 antibody	$2.89\times 10^{1}$ *g*/(*cm*^3^*h*)	Est
$\lambda_{S}$	Secretion rate of IFN-*β*	$1.386\times 10^{-8}$ *g*/(*cm*^3^*h*)	Est
$\lambda_{G}$	Secretion rate of TGF-*β*	2.8881$\times 10^{-19}$ g/(cells.h)	Est
Degradation rates of TANs & stimuli			
$\alpha_{1}$	Killing rate of N1 TANs from competition with N2 TANs	$1.5\times 10^{-8}$ *cm*^3^*cells*^−1^*h*^−1^	Est
$\alpha_{2}$	Killing rate of N2 TANs from competition with N1 TANs	$0.5\times 10^{-8}$ *cm*^3^*cells*^−1^*h*^−1^	Est
*w*_1_	Promotion strength of N1 proliferation from IFN-*β*	$1.0\times 10^{14}$ *cells*/*g*	Est
*w*_2_	Promotion strength of N2 proliferation from TGF-*β*	$1.0\times 10^{15}$ *cells*/*g*	Est
$\mu_{1}$	Decay rate of N1 TANs	$4.01\times 10^{-2}$ *h*^−1^	[23]
$\mu_{2}$	Decay rate of N2 TANs	$4.01\times 10^{-2}$ *h*^−1^	[23]
*β*	Inhibition rate of N2 antibody	$1.5\times 10^{-1}$ *cm*^3^*g*^−1^*h*^−1^	Est
$\mu_{A}$	Decay rate of N2 antibody	$2.89\times 10^{-2}$ *h*^−1^	[62], Est
$\mu_{S}$	Decay rate of IFN-*β*	$1.386\times 10^{-1}$ *h*^−1^	[63]
$\mu_{G}$	Decay rate of TGF-*β*	$2.8881\times 10^{-2}$ *h*^−1^	[64]
Tumor cells module			
*r*	Growth rate	$8.40\times 10^{-2}$ *h*^−1^	[65]
*r*_*N*_	Autocatalytic strength	1	Est
*k*	Hill-type kinetic parameter	$1.0\times 10^{6}$ *cells*/*cm*^3^	Est
*n*_0_	Carrying capacity	$1.0\times 10^{9}$ *cells*/*cm*^3^	Est
*μ_n_*	Killing rate by N1	$5.0\times 10^{-8}$ *cm*^3^*cells*^−1^*h*^−1^	Est
Diffusion coefficients			
*D*_1_	N1 TANs	$3.96\times 10^{-6}$ *cm*^2^*h*^−1^	[66]
*D*_2_	N2 TANs	$3.96\times 10^{-6}$ *cm*^2^*h*^−1^	[66]
*D*_*A*_	N2 antibody	$1.044\times 10^{-3}$ *cm*^2^*h*^−1^	[67]
*D*_*S*_	IFN-*β*	$7.668\times 10^{-2}$ *cm*^2^*h*^−1^	[68], Est
*D*_*G*_	TGF-*β*	$7.668\times 10^{-2}$ *cm*^2^*h*^−1^	[68]
*D*_*n*_	Tumor cells	$3.6\times 10^{-6}$ *cm*^2^*h*^−1^	[64]

## Results

### Dynamics of TAN-glioma in brain tumor microenvironment

**Fig 3:**
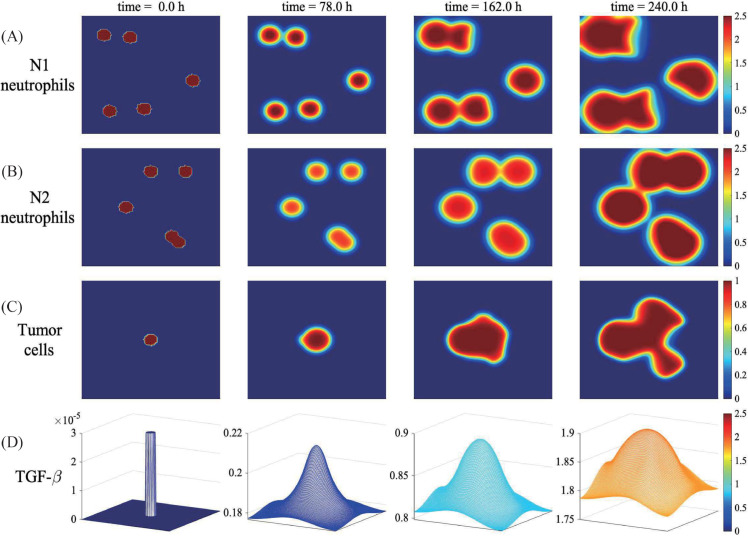
Growth pattern of a tumor via interaction with TANs. (A-D) Spatial distribution of densities of N1 TANs (A), N2 TANs (B) and tumor cells (C), and concentration of TGF-*β* (D) at t=0,78,162,240 h.

In Fig 3, we investigate the spatiotemporal dynamics of glioma cells with mutual biochemical interactions with N1/N2 TANs in the computation domain [0,1]^2^, brain tumor microenvironment, by solving the model Eqs (1)–(10). Two types of neutrophils initially distributed at a random position (first columns in Fig 3A and 3B) begin to interact with tumor cells at the center of the domain. As the tumor grows, TGF-*β* accumulates at the center (Fig 3D) due to secretion from tumor cells and diffuses through the surrounding brain TME, which enhances proliferative capacity of N2 TANs (Eq (2); Fig 3B). This activity of N2 TANs in the local TME, in turn, promotes local tumor growth (Fig 3C). Thus, the growth pattern of the tumor is not isotropic, rather immune-controlled heterogenous growth in TME.

**Fig 4:**
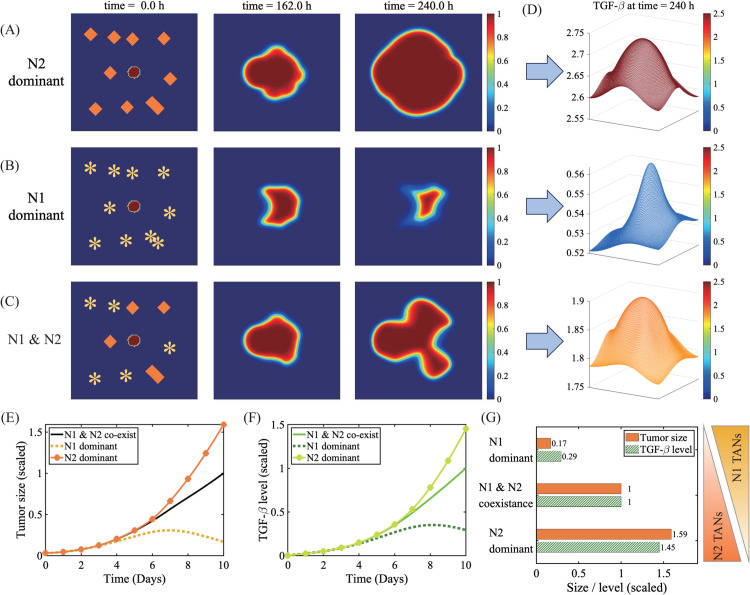
Tumor growth pattern under TME with different N1/N2 ratios. (A-C) Time courses of spatial profiles of a tumor subject to the N2-dominant (A), N1-dominant (B), and N1 & N2 coexisting (C) conditions as an initial distribution. *Asterisk=N1 TAN, diamond=N2 TAN. (D) Spatial profiles of the TGF-*β* concentration corresponding to three cases in (A) at final time. (E,F) Time evolution of the tumor size and TGF-*β* concentration in three cases in (A-C). (G) The tumor size (solid yellow) and TGF-*β* level (shaded) at the final timepoint in response to different composition rates of N1 and N2 TANs in three cases in (A-C).

The effect of different spatial distributions of the N1 or N2 TANs in TME on tumor growth patterns is more evident in Fig 4. When a tumor is surrounded by a group of N2 TAN aggregates (orange diamond in the first column of Fig 4A), the tumor grows fast (Fig 4A) with a relatively regular shape due to the positive feedback loop between the tumor and N2 TANs via TGF-*β* (Fig 4D). On the other hand, active tumor growth can be inhibited (Fig 4B) when it is embedded in a network of the classical form of neutrophils, N1 TANs (asterisks in the first column of Fig 4B). In a heterogenous brain tissue, the tumor interacts with both N1 and N2 TANs (asterisks and diamond in 1st column of Fig 4C), generating mixed growth pattern (Fig 4C) by the positive feedback loop through TGF-*β* and negative regulation of invasion and growth via IFN-*β*. Fig 4E and 4F shows time courses of the tumor size and TGF-*β* level in three cases in Fig 4A–4C. Fig 4G illustrates how the tumor size at final time is associated with the N1/N2 composition in the phenotypic spectrum of TANs. The tumor size at final time in the presence of a N1-dominant condition (yellow in Fig 4E) is decreased to 17% relative to the coexistence case (black in Fig 4E) while the tumor size subject to the N2-dominant environment (orange in Fig 4E) is increased by 59% relative to the N1/N2 coexistence case (black). These tumor-promoting effects of N2 TANs were observed in experiments, leading to poor prognosis [69].

**Fig 5:**
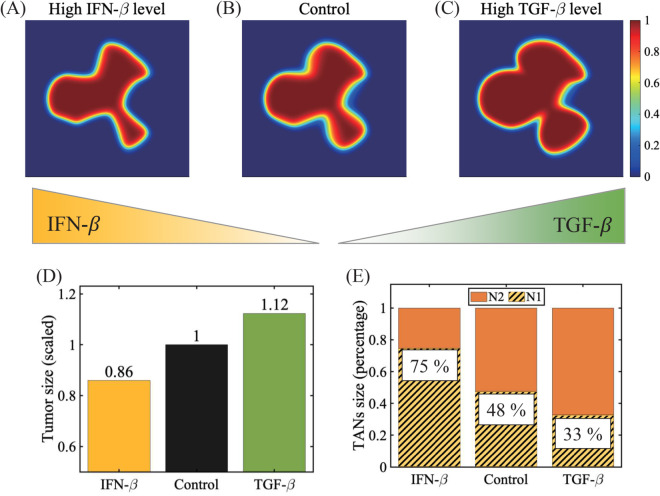
Tumor growth under different cytokine conditions. (A-C) Spatial profile of a tumor at final time in the IFN-*β*-rich condition (A), control (B), and TGF-*β*-prevalent environment. (D,E) The tumor size (D) and relative percentage of TANs at final time in three cases in (A-C). Parameters: higher secretion rates of IFN-*β* and TGF-*β* used: $\lambda_{S}=100\;*\;\lambda_{S}^{†}$ in (A), $\lambda_{G}=100\;*\;\lambda_{G}^{†}$ in (C) relative to the base parameters $\lambda_{S}^{†},\lambda_{G}^{†}$ in (B). Initial spatial distribution of densities of the N1 and N2 TANs is same as one in Figs 3 and 4.

Fig 5 depicts the growth pattern of a tumor under two key cytokines (IFN-*β* and TGF-*β*) conditions relative to the control case with base production rates of IFN-*β* and TGF-*β* ($\lambda_{S}^{†},\lambda_{G}^{†}$). When a tumor is embedded in the IFN-*β*-rich TME, tumor invasion and growth are inhibited (Fig 5A; $\lambda_{S}=100\;*\;\lambda_{S}^{†}$) relative to the control (Fig 5B; $\lambda_{S}^{†}$) resulting in the smaller tumor size (yellow, Fig 5D), due to more activated N1 TANs (Fig 5E). On the other hand, a tumor under the TGF-*β*-prevalent condition invades and grows faster (Fig 5C; $\lambda_{G}=100\;*\;\lambda_{G}^{†}$) relative to the control (Fig 5B; $\lambda_{G}^{†}$). The larger number of N2 TANs (67%, Fig 5E) compared to the N1 TAN population essentially drives this faster growth (green bar in Fig 5D).

**Fig 6:**
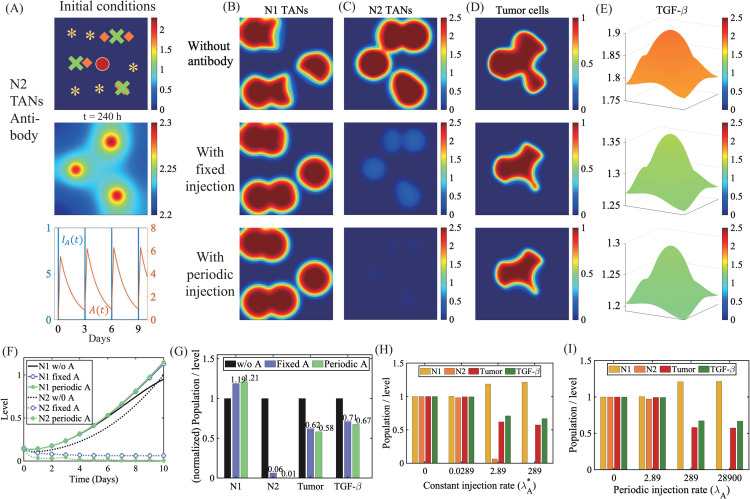
Dynamics in response to antibody against N2 TANs. (A) (upper panel) Initial distribution of variables, (middle panel) spatial profile of antibody against N2 TANs at final time in response to constant injection ($\lambda_{A}^{*}$), and (lower panel) a time course of the antibody concentration (orange; *A*(*t*)) and periodic injection function (blue; *I*_*A*_(*t*)). *Asterisk=N1 TANs, diamond = N2 TANs, X= antibody injection site. (B-E) Spatial profiles of densities of N1 (B) and N2 (C) TANs, and tumor (D), and TGF-*β* concentration (E) at final time in control (1st row), constant injection (2nd row), and periodic injection (3rd row), respectively in (A). (F) Time courses of the populations of N1 and N2 TANs in three cases in (B-E). (G) Normalized populations of N1/N2 TANs and tumor cells, and TGF-*β* level at final time in three cases in (B-E). (H,I) Normalized populations of N1/N2 TANs, and tumor cells and TGF-*β* level at final time in response to constant injection of the antibody for various rates ($\lambda_{A}^{*}=0,0.0289,2.89,289$; (H)) and periodic injection ($\lambda_{A}(t)=100\lambda_{A}^{*}\sum_{j=1}^{4}I_{\left[t_{j},t_{j}+\tau \right]}$, τ=1 h).

In Fig 6, we investigate the anti-tumor effect of the antibody against N2 TANs. Initial distribution of variables is shown in the upper panel of Fig 6A where the antibody was injected at random positions (green cross marker, upper panel) close to N2 TANs near the primary tumor (circle in upper panel). For the injection term in Eq (3), we take either a constant injection rate $\lambda_{A}(t)=\lambda_{A}^{*}$ or a periodic injection over the time intervals $\left[t_{j},t_{j}+\tau \right]$ ($j=1,\ldots ,N_{inj}$) as follows:

$$\lambda_{A}(t)=100\lambda_{A}^{*}\;I_{A}(t),\;\;\;\;I_{A}(t)=\sum_{j=1}^{N_{inj}}I_{\left[t_{j},t_{j}+\tau \right]}$$ (11)

where the indicator function $I_{\left[t_{j},t_{j}+\tau \right]}$ gives 1 over the time intervals $\left[t_{j},t_{j}\;+\;\tau \right]$ ($j=1,\ldots ,N_{inj}$) and zero otherwise. Here, we take $\tau =1\;h,N_{inj}=4$. The lower panel in Fig 6A shows time courses of the periodic injection function *I*_*A*_(*t*) and corresponding antibody concentration (orange; *A*(*t*)). Fig 6B–6E shows the spatial profiles of the densities of N1 TANs (Fig 6B), N2 TANs (Fig 6C), and tumor cells (Fig 6D) and concentration of TGF-*β* (Fig 6E) at final time (t=240 h) in response to antibody injection (middle & lower panels) relative to control case (upper panel). The antibody can effectively suppress the spread and activities of N2 TANs (Fig 6C, Fig 6F). This in turn reduces the tumor-promoting effect of N2 TANs (Eq (5)) and blocks activities of tumorigenic TGF-*β* (Fig 6E, Fig 6G), shaping anti-tumorigenic environment and leading to the smaller tumor (~38% reduction; third column, Fig 6G). We also examined how the strength of the antibody injection alters dynamics of TANs and affects tumor growth. Fig 6H and 6I summarizes the changes in the tumor sizes in response to the antibody injection at various rates ($\lambda_{A}^{*}=0,0.0289,2.89,289$) for both constant and periodic injections, illustrating consistent anti-tumor efficacy in both cases. As $\lambda_{A}$ is increased, the N2 population is decreased but the N1 population is increased, resulting in a decrease in the TGF-*β* level and finally the reduced glioma size.

**Fig 7:**
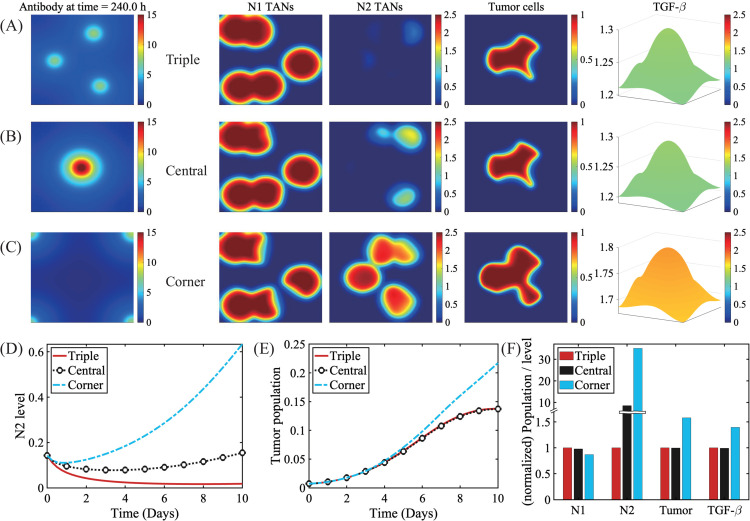
Effect of injection locations on N1/N2 dynamics and tumor growth. (A-C) Spatial profile of antibody concentration. densities of N1 TANs, N2 TANs, and tumor cells, and TGF-*β* concentration at final time when antibody was injected at the N2 TAN sites (A), in the center (B), and on the four corners (C). The total amount of injected antibody was the same in three cases. (D,E) Time courses of the N2 TAN population (D) and tumor population (E) in three cases in (A-C). (F) Normalized populations of N1/N2 TANs and tumor cells, and level of TGF-*β* in three cases in (A-C).

In Fig 7, we investigate the effect of spatial injection sites on anti-tumor efficacy. The tissue composition in the brain varies significantly, affecting diffusion coefficient, and in oder to see the direct effect of injection sites, we set $D_{A}=D_{A}^{*}/100$. We compare three cases where the antibody was injected in the N2 sites (Fig 7A, targeting therapy, triple), the center of the tumor (center of the computational domain, Fig 7B, central), and four corners of the domain (Fig 7C, corner) while the total amount of antibody is fixed. Antibody injected into the tumor center does not directly kill tumor cells but diffuses to the neighboring N2 TAN sites, thus suppressing N2 TANs, increasing N1 TAN activities, and eventually killing tumor cells in the neighborhood. However, the lower level of antibody at the N2 sites after diffusion leads to mild suppression of N2 TANs (circle dashed, Fig 7D) compared to the direct targeting (red solid, Fig 7D). In this case, the survived N2 TANs (3rd panel, Fig 7B) does not have significant impact on tumor promotion (Fig 7E) because of the non-overlapping, localized location. When the antibody is injected in the far field, it fails to suppress N2 TANs near the tumor (3rd panel, Fig 7C; blue dashed, Fig 7D) and the tumor grows faster in communication with N2 TANs (blue dashed, Fig 7E). Fig 7F shows the summary of normalized populations of N1 TANs, N2 TANs, tumor cells, and TGF-*β* concnetration at final time. This illustrates that anti-tumor efficacy is not effective when the injection site is farther from the N2 TANs despite the equivalent total amount of the antibody drug.

**Fig 8:**
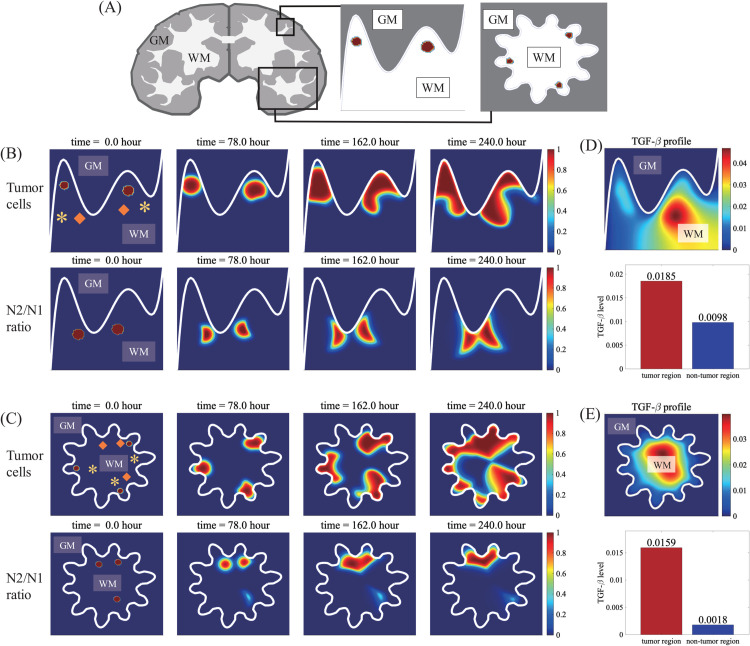
Glioma invasion in the presence of complex geometry with white and gray matter. (A) Illustration of a human brain with tissue type labels for two representative brain regions in the presence of white (WM) and gray (GM) matter. (B-C, upper panel) Spatial profiles of the growth patterns of glioma cells starting from a white matter region surrounded by gray matter described in different brain regions described in (A): a single wrinkle (B), a star-shaped area (C). *Asterisk=N1 TANs, diamond = N2 TANs. (B-C, lower panel) Spectral distribution of N1 and N2 TANs (N2-to-N1 ratio) corresponding to times in the upper panel. (D,E) Spatial profile (upper panel) of TGF-*β* and relative spatial composition in the tumor region and other region (lower panel) at final time (t=240 h), corresponding to (B) and (C), respectively.

While the role of neutrophils in glioma dynamics has been underestimated [70], increasing evidence underscores the importance of the significant dual role of TANs in cancer progression [71–73] and the ratio of neutrophils to lymphocytes (NLR) in glioma TME as diagnostic tools [74]. In Fig 8 we investigate the growth pattern of glioma cells embedded in various physical environments such as gray matter and white matter. Thus, our computational domain ($\Omega =[0,1{]}^{2}$) is divided into two smooth sub-domains: gray matter ($\Omega^{+}$) and white matter ($\Omega^{-}$), $\Omega =\Omega^{+}\dot{⋃}\Omega^{-}$, with the interface Γ. Motility constants of cells and diffusion coefficients of cytokines in the gray matter ($D_{i}^{G}$) are significantly lower than ones in white matter ($D_{i}^{W}$) as observed experimentally [75–79], *i.e.* $D_{i}^{G}=$ $10^{-6}D_{i}^{W},$ i=1,2,A,n,S,G. As a tumor grows in white matter, the pro-tumorigenic factor, TGF-*β*, diffuses through the white matter (Fig 8E), enhancing the activity of N2 TANs and suppressing N1-mediated tumor cell killing (Fig 8B and 8C). Here, the spatial profiles of the N2 to N1 ratio (*SN*21*R*) distribution at t=0,78,162,240 h are shown in the lower panel of Fig 8B and 8C. Here, the *SN*21*R* value at given position x and time *t* was calculated by the relative balance with a scaling factor *ε* to avoid the ∞ value where $N_{1}(x,t)=0$, *i.e.* we define

$$SN21R(x,t)=\frac{N_{2}(x,t)}{\epsilon +N_{1}(x,t)}.$$ (12)

Here, we set ϵ=0.01. This TAN-induced asymmetry in the local tumor region, either faster growth in the N2-rich environment or suppression under the N1-favorable toxic condition, leads to anisotropic tumor invasion and growth (last column in Fig 8B and 8C). For example, two sets of tumor mass initiated at the two different locations in the valley of a single wrinkle grow in a nonlinear fashion at the faster rate in N2-rich spots (lower panel in Fig 8B) along the white-gray matter interface (Γ, white curves in the upper panel in Fig 8B) in the middle and slower growth in the N1-dominant area (Fig 8B). Three groups of tumor mass enclosed in a different valley in star-shaped white matter (upper panel) follow the similar N2-mediated growth pattern, but these glioma cells infiltrate the inner white matter (Fig 8C).

**Fig 9:**
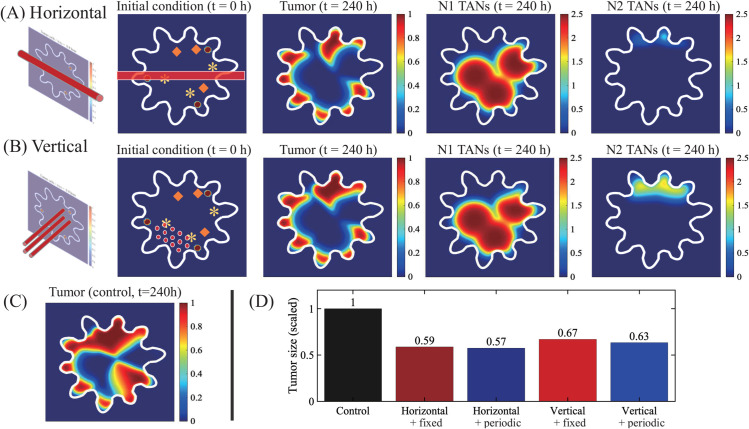
Effect of N2 antibody injection on glioma growth (A-B) Diagram of BV distribution, Initial distribution of variables, and spatial profiles of the densities of tumor cells, N1 TANs, and N2 TANs at final time (t=240 h) in response to N2 antibody injection through BVs in horizontal (A), vertical (B) BV geometries. (C) Spatial profile of the tumor density at final time in control (without antibody). (D) Tumor size in response to N2 antibody injection (control, horizontal+fixed, horizontal+periodic, vertical+fixed, and vertical+periodic).

In Fig 9, we investigate the anti-tumor efficacy of N2 antibody injection through BVs in the TME from constant or periodic injections as in Fig 6. The BBB is a major hurdle of drug delivery in brain [80] but it can be remodeled with nonlinear functionalities, causing a reshaped vasculature called the blood-tumour barrier (BTB), due to the biomechanical pressure as brain tumor grows [80] and/or a certain type of therapeutic approaches such as oncolytic virus (OV) therapy. For instance, OVs can change biophysical properties of blood vessels in gliomas, causing hyperpermeability for improved immune cell infiltration and drug entry [81], and complex changes such as angiogenesis [82]. The BTB then can lead to a local accumulation of drugs near BVs with heterogeneous perfusion [83–86], thus affecting on immunotherapy [87,88]. For the test, the geometry of BVs (1st column) and initial conditions (2nd column) were given in Fig 9A and 9B, for horizontal and vertical BV cases, respectively. As N2 antibodies penetrate the brain tissue from BVs that cross the horizontal center of the computational domain, tumor growth is significantly inhibited (3rd column, Fig 9A) compared to the control case (Fig 9C) from more activated N1 TANs (4th column in Fig 9A) and decreased activities of N2 TANs (5th column in Fig 9A). This resulted in the significant reduction in the tumor size compared to the control case (∼41% and 43% reduction, for constant and periodic injections, respectively; Fig 9D). However, we note that some of tumor cells, especially ones in the inner part of white matter valley near gray matter still survive from this anti-tumor strategies due to limited access from N1 TAN activities (4th column, Fig 9A). The spatial profiles of densities of tumor cells, N1 TANs, and N2 TANs in response to the N2 antibody injection in vertical geometry is also shown in Fig 9B. Anti-tumor efficacy is a bit lower than the horizontal case due to active tumor cell killing near BVs in the lower part of the domain but with a relatively low effect in the distant field in the upper domain (∼33% and 37% reduction for constant and periodic injections, respectively, Fig 9D). Thus, the physical distribution of BVs as well as permeability of BBB plays a significant role in tumor cell killing.

### Sensitivity analysis

In order to investigate the sensitivity of some parameters in the model, we performed a sensitivity analysis on twenty six parameters to examine correlations with key variables of the PDE model in Eqs (1)–(10). For sensitivity analysis, a large data set from computer simulations was first collected from the PDE model with the set of randomly selected parameters. Based on the sensitivity analysis suggested by Marino et al. [89] and MATLAB files (http://malthus.micro.med.umich.edu/lab/usadata/) and simulation data, we used general Latin hypercube sampling (LHS) scheme and partial rank correlation coefficient (PRCC) for twenty six parameters ($D_{1},D_{2},D_{A},D_{S},D_{G},D_{n},r_{1},r_{2},K_{1},K_{2}$, $\alpha_{1},\alpha_{2}$, $\mu_{1}$, $\mu_{2}$, $\lambda_{A},\mu_{A}$, $\lambda_{S}$, $\mu_{S}$, $\lambda_{G},\mu_{G}$, *r*,*r*_*N*_, $k,n_{0},\mu_{n}$, *β*) in the model for all model variables ($N_{1},N_{2},A,S,G,n$). We have chosen a range of each parameter and divided them with 1,000 uniform-length subintervals, and calculated PRCC values and p-values at t=1,50,100 hour. Each PRCC value lies on the interval [–1,1] with a sign indicating the positive (or negative) correlation.

**Fig 10:**
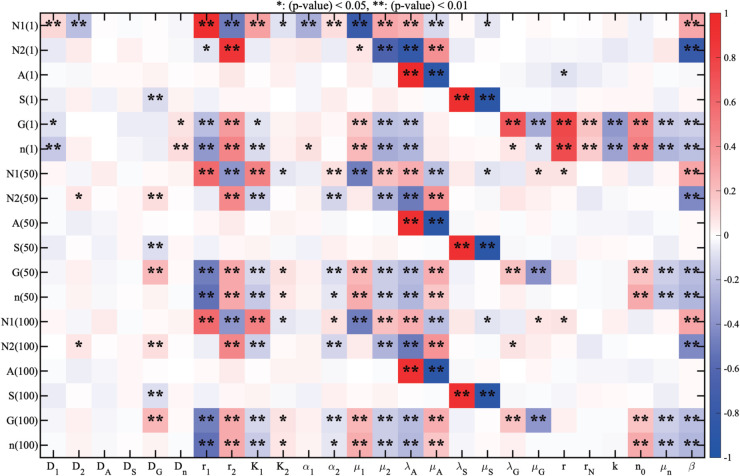
Sensitivity analysis. LHS scheme and PRCC are used to analyze the PDE model in Eqs (1)–(10) at time t=1,50,100 hour with a sample size 1,000 [89]. Each colored box indicates the PRCC value of each variable for parameters in the model: red (or blue) represents the positive (or negative) correlation.

Sensitivity analysis results are summarized in Fig 10. Color of each cell indicates either positive (red) or negative (blue) correlation of the parameter to cell populations (N1/N2 TANs, and tumor cells) and molecule levels at t=1,50,100 h. From now on, the population of N1 TANs at time *t* was calculated by integrating the density of N1 TANs over the computational domain Ω: ${\hat{N}}_{1}(t)=\int_{\Omega }N_{1}(x,t)\;dx$. In a similar fashion, we define the populations of cells and levels of main molecules in the model: N2 TANs (${\hat{N}}_{2}(t)=\int_{\Omega }N_{2}(x,t)\;dx$), antibody ($\hat{A}(t)=\int_{\Omega }A(x,t)\;dx$), tumor cells ($\hat{n}(t)=\int_{\Omega }n(x,t)\;dx$), IFN-*β* ($\hat{S}(t)=\int_{\Omega }S(x,t)\;dx$), TGF-*β* ($\hat{G}(t)=\int_{\Omega }G(x,t)\;dx$). The star sign in each cell denotes p-value associated with the PRCC value: a single star for (*p*–*value*)<0.05 and double star for (*p*–*value*)<0.01. Based on these statistical analysis results, we conclude the following: (i) The parameters associated with movement of N1 TANs (*D*_1_), proliferation of N1 TANs ($r_{1},K_{1}$) and suppression of N2 TANs ($\mu_{2},\beta ,\lambda_{A}$) are positively correlated with the N1 TAN population while movement of N2 TANs (*D*_2_), inhibition parameters of N1 TANs ($\alpha_{1},\mu_{1}$) and promotion rates of N2 TANs ($r_{2},K_{2},\mu_{A}$) have a negative correlation with the N1 TAN population. Especially, *r*_1_ (or $\mu_{1}$) has a very strong positive (or negative) correlation with the N1 TANs population. (ii) The parameters $r_{2},\mu_{A}$ have a positive correlation with N2 TANs but $\mu_{2},\beta ,\lambda_{A}$ have a negative correlation with the N2 population. (iii) The tumor cell population shares a similar set of parameters with N2 TANs for positive and negative correlations, except *n*_0_. The carrying capacity *n*_0_ of tumor cells is positively correlated with tumor population as expected. (iv) The source term (or decay rate) for each cytokines (N2 antibody (*A*), IFN-*β* (*S*), and TGF-*β* (*G*)) has a strong positive correlation with the main corresponding variable. For example, the TGF-*β* source ($\lambda_{G}$) is positively correlated with the TGF-*β* concentration and the decay rate of IFN-*β* ($\mu_{S}$) is negatively correlated with the IFN-*β* level at given time points. See also S4 Text for sensitivity analysis of the basic unit (ODE model) and S5 Text for sensitivity of 6 key parameters ($r_{1},r_{2},\alpha_{1},\alpha_{2},\lambda_{S},\lambda_{G}$) to main variables in the dynamics of the PDE model.

### Therapeutic approach

**Fig 11:**
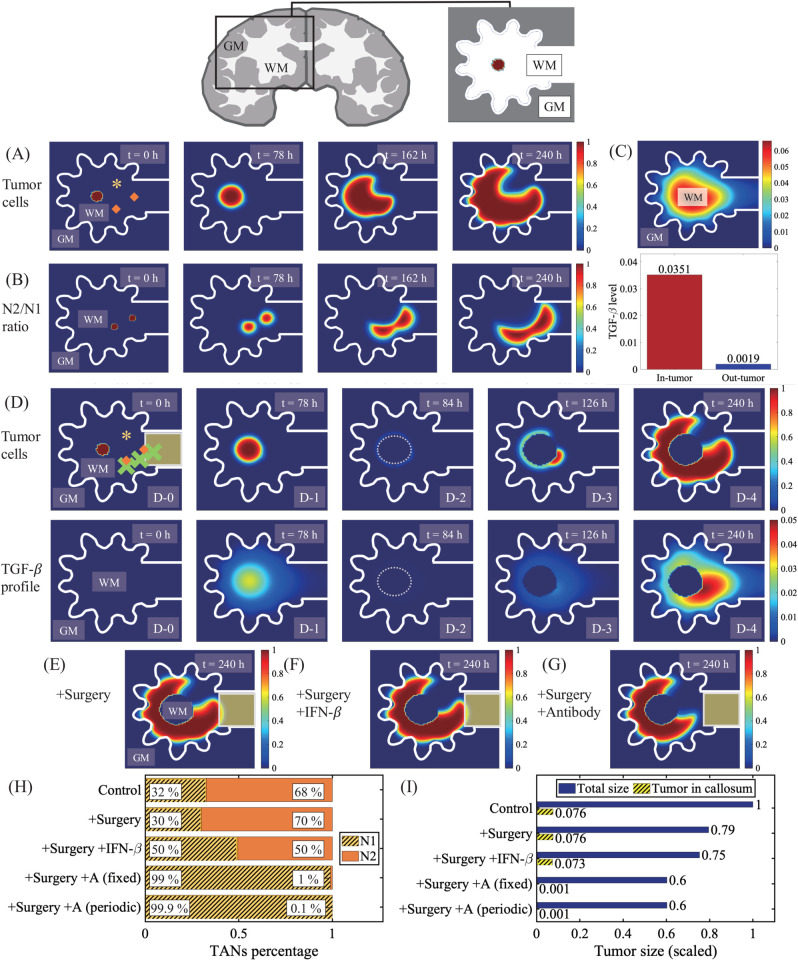
Glioma cell migration through corpus callosum (CC) after surgical resection. (top) Illustration of a human brain with a tumor near CC in the center of computation domain (box). (A-B) Growth pattern of a glioma (A) and spatial distribution of the N2/N1 ratio (B) at t=0,78,162,240 h in the area near corpus callosum. Tumor growth began in the white matter area surrounded by gray matter. (C) Spatial profile of the TGF-*β* concentration (upper panel) and TGF-*β* levels in tumor and remaining regions (lower panel) corresponding to (A-B) at final time. (D) Spatial profiles of the tumor cell density and TGF-*β* concentration before (t=0,78 h) and after (t=84,126,240 h) surgical resection of glioma in the center at t=84 h. (E-G) Spatial profile of the tumor density at t=240 h in the case of surgery (E), surgery+IFN-*β* (F), and surgery+N2 antibody (G). (H) Relative populations of N1 (yellow stripes) and N2 (orange) TANs in control, surgery, surgery+IFN-*β*, surgery+N2 antibody. (I) Populations of all tumor cells (blue) and invasive tumor cells in CC (stripes) corresponding to cases in (H). Asterisk(*)=N1 TANs, diamond = N2 TANs. *WM = white matter, GM = gray matter.

A conventional therapy of GBM is surgical resection of the tumor mass though this typically leads to cellular infiltration of survived, invisible glioma cells to surrounding brain tissue and local metastasis. Surgery also results in faster growth via interactions with other cells in brain such as astorcytes and microglia [46]. Cellular invasion of glioma cells through CC, a thick bridge of dense myelinated white-matter fibers connecting the two sides of hemispheres of the brain [90], often presents challenges in surgical resection due to fast migration [91] and regrowth of glioma cells associated with poor prognosis [49–51]. In Fig 11, we investigate the role of TANs in regulation of glioma invasion near the CC. When a brain tumor grows in the neighborhood of the callosum commissure (Fig 11A), the active spreading through the corpus callosum is assisted by prevalent N2 TANs near the edge of the CC due to localized, high SN21R levels (Fig 11B) along with faster diffusion of TGF-*β* (Fig 11C). Proximity to the CC increases the potential of infiltration, which is observed in a significant number (∼25%) of glioblastoma patients at diagnosis [92]. This infiltration of glioma cells through the CC eventually leads to formation of the secondary tumor in the opposite side of the brain, butterfly GBM [92,93]. These results overall indicate the SN21R index in addition to information from images from conventional approaches (MRI, CT, X-ray) and pathological data can be used to detect the onset of glioma and CC infiltration in brain and to target the fastest growing tumor area as well as recurrence of GBM for therapy as other researchers suggested [74].

Surgical removal of a glioma mass at t=84 h may seem to be effective (D-2 in Fig 11D) but remaining glioma cells begin to grow back on the periphery of the resected area (D-3 in Fig 11D) as observed in experiments [46–48], eventually repopulating the tumor in the left-hemisphere (D-4 in Fig 11D) and increasing an invasive potential toward the right hemisphere through the neighboring CC. When IFN-*β* is injected after surgery, the relative portion of N1 TANs is increased (30%→50%; Fig 11H), and the whole tumor population (Fig 11F and 11I) and the population of invasive glioma cells in the CC is slightly reduced (Fig 11I). In contrast, injected N2 antibody in TME promotes the strong $N_{2}\to N_{1}$ phenotypic transition (Fig 11H), which leads to inhibition of tumor growth near the CC after surgery (Fig 11G) and effectively prevents migration into the contralateral hemisphere, lowering potential of butterfly gliomas (Fig 11I).

### Hybrid multi-scale model approaches

**Fig 12:**
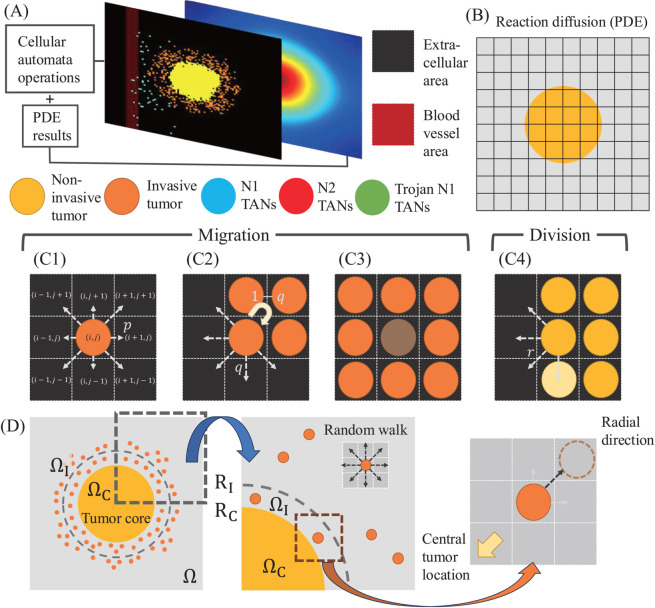
Schematic of the hybrid model. (A) Structure of the hybrid model where BV structure, reaction-diffusion, and discrete five types of cells (non-invasive glioma cells, invasive glioma cells, N1 TANs, N2 TANs, Trojan N1 TANs) are immersed in a regular grid. Phenotypic change between N1 and N2 TANs are based on the level of IFN-*β* and TGF-*β* levels at the local site ($x_{i},y_{i}$). (B) Reaction-diffusion equations of concentrations of TGF-*β*, IFN-*β*, and antibody are solved on the regular grid. (C) Migration (C1-C3) and proliferation (C4) of *i*-th cell are determined based on bio-physical and bio-chemical conditions in the neighborhood of the local cell site ($x_{i},y_{i}$). (D) Rules of migration directions of invasive tumor cells in physical environment and biochemical conditions. Invasive tumor cells in the close outer rim ($\Omega_{I}$) migrate away from tumor core ($\Omega_{C}$) while they take a random walk outside this region.

While PDE models can represent the spatio-temporal dynamics of the system at the macroscale, a hybrid model can be adapted to track down the details in the dynamics at the cellular scale such as cellular movement, proliferation, and cell-cell interactions in the presence of detailed brain structure of white and gray matter, and corpus callosum. Hereafter, we consider a hybrid-approach. Consider brain tissue Ω with a GBM tumor initially occupying a sphere in the center and a blood vessel on the left in the microenvironment (sources of immune cells such as TANs). Our hybrid multiscale model on our computation domain Ω includes several components: (1) cell-based model for growth/migration dynamics of five types of cells (non-invasive glioma cells, invasive glioma cells, N1 TANs, N2 TANs, Trojan N1 TANs), (2) reaction-diffusion PDE model of extracellular key diffusible molecules (IFN-*β* and TGF-*β*), and (3) blood vessel structure. To elaborate the movements of cells at the cellular scale, a cell-based rule mechanics such as cellular automata (CA) [54,55] was used. Glioma cells either proliferate or migrate under a biophysical condition. A proliferating glioma cell on the surface of the tumor core becomes an invasive cell and migrates away when the physical space in the neighborhood (eight spots) is available in the migration direction. The migration direction of the invasive glioma cell is influenced by random motility and chemotaxis based on local concentrations of chemoattractants in the reaction-diffusion model. On the other hand, dynamics of IFN-*β* and TGF-*β* in the reaction-diffusion model depend on individual cellular components; secretion of TGF-*β* by glioma cells. Thus, growth and invasion of glioma cells can affect concentrations of diffusible molecules in the PDE system and these changes are incorporated into the behavior of other TANs (N1, N2, Trojan N1 TANs) from a BV due to the spatial and temporal heterogeneity of TGF-*β* levels. For therapeutic purposes, Trojan TANs are introduced from the BV to the microenvironment to effective tumor cell killing. The neutrophil-mediated delivery system recognizes inflammatory signals such as IL-8 and CXCL1/KC [94,95] after conventional surgery and transport chemo-drugs (nanoparticles) to glioma cells. Then, inflammatory cytokines mediate neutrophil activation in TME, leading to the disruption of TANs and release of NETs and liposomes [96], thus delivering nanoparticle into the glioma cells. In our framework, we assume a Trojan N1 TAN kills glioma cells in the neighborhood under physical contact conditions instead of adapting the detailed spatio-temporal dynamics of nanoparticles from Trojan N1 TANs. See Fig 12 for schematic diagram of the hybrid multi-scale model showing the appropriate scales involved.

The computational algorithm:

**Step 0** Initialization.
  **Step 0.1** Set geometry of BV and CC when applied (Fig 12A).
  **Step 0.2** Set a rectangular grid for reaction-diffusion (PDE) algorithm that determines concentrations of IFN-*β*, TGF-*β*, and antibody, and set the initial condition (Fig 12B).
  **Step 0.3** Initialize cell-based component (cellular automata) by randomly placing cells in the tumor core domain and placing N1 TANs, N2 TANs, Trojan N1 TANs in given BV. Initially all tumor cells are non-invasive tumor cells. Initialize the cell cycle time.
  **Step 0.3** Use these non-invasive tumor cells to calculate the radius *R*_*c*_ of the tumor core and determine boundaries of the tumor (Fig 12D).
**Step 1** Locate all cells (non-invasive tumor cell, invasive tumor cell, N1 TAN, N2 TAN, Trojan N1 TAN) in the neighborhood $N_{i}$ of cell *i*.
**Step 2** Determine Go or Grow process for tumor cell *i* on the periphery ($\Omega_{I}$, Fig 12D) of the growing tumor
  **Step 2.1** The non-invasive tumor cell *i* on the periphery of tumor core ($\Omega_{C}$) becomes an invasive tumor cell with a probability *q* (Fig 12C1 and 12C2) and go to [Step 3].
  **Step 2.2** Otherwise, allow the non-invasive tumor cell to grow with a probability 1–*q* and go to [Step 4].
**Step 3** Translation of cells (migration).
  **Step 3.1** Identify cells from each of the neighbor cells $N_{i}$ found in [Step 1].
  **Step 3.2** Find all available empty spot in the neighborhood $N_{i}$ of cell *i* (Fig 12C1 and 12C2).
  **Step 3.3** Move the invasive tumor cells in $\Omega_{I}$ based on the gradient of TGF-*β* concentration (negative chemotaxis, radial direction). Move the invasive tumor cells outside $\Omega_{I}$ in a random location among available spots with a probability *p* using a random number.
  **Step 3.4** Move TANs (N1 TANs, N2 TANs, Trojan N1 TANs) in the direction of the tumor based on the gradient of TGF-*β* concentration (*chemotaxis*).
**Step 4** Divide cells
  **Step 4.1** Check the cell cycle of the cells (time after last division)
  **Step 4.2** If they reach the end of their cell cycle (8 hours), determine if the cell is enclosed by other cells (Fig 12C3).
  **Step 4.3** Divide cells in the absence of physical constraint. Axis of division is determined by random selection among available spots in the neighborhood $N_{i}$ with a probability *r* (Fig 12C4).
  **Step 4.4** Reset the cell cycle clock after cell division from [Step 4.3].
**Step 5** Update the N1 and N2 TAN status based on IFN-*β* and TGF-*β* levels.
**Step 6** Kill the tumor cells by removing the tumor cells from computation domain when N1 TANs or Trojan N1 TANs makes a contact in the neighborhood $N_{i}$. Update the radius *R*_*c*_ of the tumor core and *R*_*I*_ of the invasive rim.
**Step 7** Remove all tumor cells within a radius in the case of surgery and additional tumor cells in the resected area in the case of extended surgery.
**Step 8** Update the levels of IFN-*β*, TGF-*β*, and antibody solving reaction-diffusion equations in Eqs (3), (6), (7) (Fig 12B).
**Step 9** Go to [Step 1].

**Fig 13:**
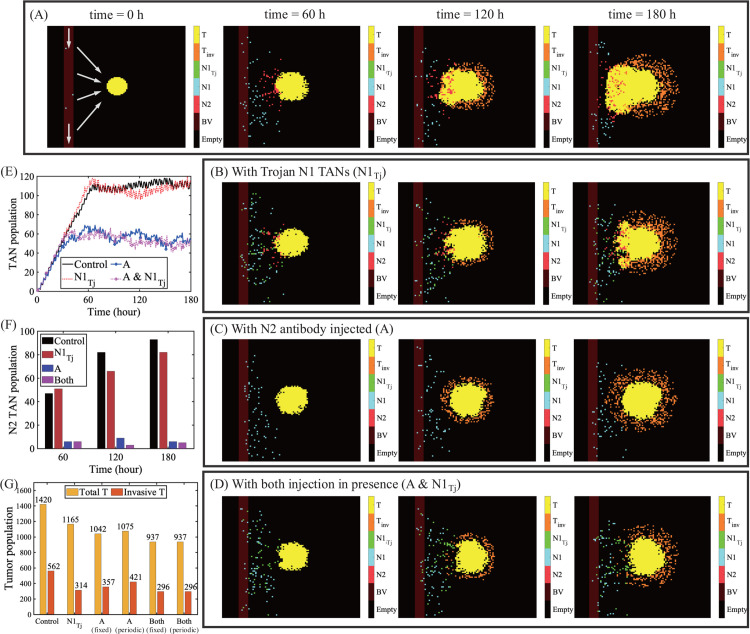
Anti-tumor efficacy of Trojan N1 neutrophil injection: Hybrid model. (A) Dynamics of proliferation and infiltration of noninvasive (yellow) and invasive (orange) glioma cells at t=0,60,120,180 h in the presence of N1 and N2 TANs from a BV on the left. (B-D) Spatial profiles of glioma cells in response to immune attack from Trojan N1 TANs (green) alone (B), N2 antibody injection (C), and Trojan N2 TANs+antibody injection (D), from the BV at t=60,120,180 h. (E) Time courses of populations of N1 and N2 TANs in response to control, Trojan N1 injection ($N1_{T_{j}}$), antibody injection (*A*), and Trojan N1 TANs+antibody injection ($A\&N1_{T_{j}}$). (F) Changes in the N2 TAN population at t=0,60,120,180 h in four cases in (E). (G) Tumor cell populations (total population (yellow bar), invasive population (red bar)) at final time in four cases in (E).

In Fig 13, we investigate the effect of N2 antibody and combined therapy (N2 antibody+ Trojan N1) on tumor growth and infiltration. A GBM is located at the center of the computational domain with BV on the left and the tumor cells begin to grow initially from the center. N1 TANs from the BV chemotactically respond to the upgradient of TGF-*β* (∇G) and migrate toward the source, the tumor. However, these N1 TANs (blue dots) transit to N2 TANs (red dots) as they get closer to the tumor due to tilted immuno-balance (up-regulation of TGF-*β* and down-regulation of IFN-*β*) in the TME (Fig 13A). This prevalence of N2 TANs in the TME not only protects the tumor cells from immune attack but actually promotes proliferation of tumor cells, angiogenesis and radiotherapy (RT) resistance [69]. When Trojan N1 cells are supplied through the BV, these immune active cells are able to penetrate the tumor and kill glioma cells (Fig 13B), effectively reducing both invasive and non-invasive tumor cells (Fig 13G). In a similar fashion, the injection of the N2 antibody leads to the phenotypic transition from N2 to N1 TANs (Fig 13E and 13F) and higher N1-mediated cell killing (Fig 13C and 13G). When these two strategies are combined, both exogenous Trojan N1 TANs and converted endogenous N1 TANs effectively get an access to tumor by removing the immune shield of N2 TANs (Fig 13D), leading to synergistic killing of both invasive (∼47% reduction) GBM cells and the whole population (∼34% reduction) (Fig 13G).

**Fig 14:**
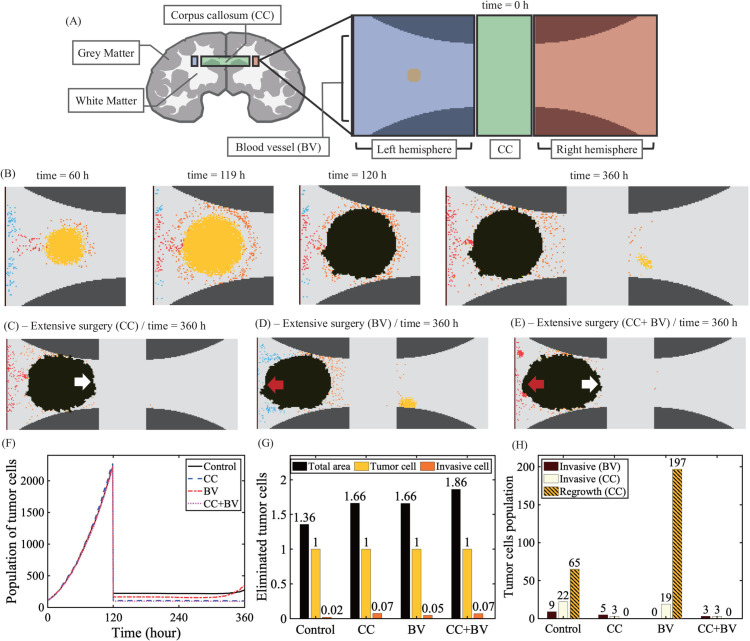
Eradication of glioma cells in both hemispheres after surgical resection of the GBM core. (A) Schematic of the computation domain consisting of the left ($\Omega_{-}$) and right ($\Omega_{+}$) subdomains that are connected by the CC ($\Omega_{0}$) with white matter bundles, creating a fast moving track. (B) Spatial profiles of the tumor core (yellow) and invasive tumor cells (orange) at t=60,119,120,360 h in response to optimal surgical resection of primary tumor core at t=120 h based on MRI images, reflecting tumor core. Infiltrative glioma cells are survived from the surgery and migrate into the other region in the opposite hemisphere through CC and begin to proliferate, forming the secondary tumor mass, local metastasis. (C-E) Spatial distribution of tumor cells at t=360 h in response to surgical resection (extended surgery, ES) of a larger domain near the entrance of CC (C), left side of the tumor core near BV (D) and both areas (E) at t=120 h. (F) Time courses of tumor populations in four cases in (B-E). (G) Area, relative populations of tumor cells and invasive tumor cells that were removed in four cases in (B-E). (H) Population of infiltrative tumor cells along BV (brown), infiltrative tumor cells in the right hemisphere (white), and recurrent tumor cell population in the right hemisphere (comb lines) in four cases in (B-E).

The special white matter bundles within CC connecting the left and right hemispheres of brain provide a platform of fast migration of glioma cells from one side to another, lowering patient quality-of-life [51]. To investigate the effect of cellular infiltration of glioma cells through CC after surgery on overall anti-tumor efficacy, we consider a computational domain (Ω) consisting of the left ($\Omega_{-}$) and right ($\Omega_{+}$) subdomains that is connected by CC ($\Omega_{0}$): $\Omega =\Omega_{-}\cup \Omega_{0}\cup \Omega_{+}$. See Fig 14A for a schematic of the computation domain. Here, we assume that the main tumor mass is surgically removed based on MRI images at t=120 h. The black mass in Fig 14B–14E) represents the removed area by surgery. The red and white arrows in Fig 14C–14E) indicate the location of extensive surgical resection of tumor in tissue close to CC (white arrow) and BV (red arrow). Due to relatively low resolution and technical difficulties, it is not possible to remove infiltrative individual glioma cells outside the *visible* tumor core [18,41,42,46,97]. Fig 14B shows the spatial distribution of glioma cells and TANs in $\Omega_{-}$ (t=60,119,120,360 h) and $\Omega_{+}$ (t=360 h). While the surgical resection of the core eliminates the majority of tumor cells (Fig 14F), a small fraction of invasive glioma cells are not resected and survive (orange, control; Fig 14G) and infiltrate the surrounding tissue (Fig 14B). Some of these infiltrative cells penetrate and migrate along the arrow CC, white matter fiber bundles, and eventually begin to proliferate again (yellow) on the other hemisphere, forming the secondary tumor mass. In the model, without surgery of the tumor mass on $\Omega_{-}$, the active regrowth of tumor at the opposite site in $\Omega_{+}$ leads to butterfly glioblastoma. Thus, an optimal surgical resection of the primary GBM before invasion through the CC may lead to better clinical outcomes. Here, we test the effect of the surgical resection of the tumor mass in larger area near CC and BV on local regrowth from the infiltrative glioma cells in the other hemisphere. Fig 14C–14E shows the spatial distribution of tumor cells on both sides at t=360 h in response to more extensive surgical removal of the tumor tissue near the entrance of CC (Fig 14C), left side of the tumor core near BV (Fig 14D) and both areas (Fig 14F). The surgical resection of the brain tissue near the CC (2nd column, Fig 14G) essentially blocked the cellular migration of glioma cells to the other hemisphere (Fig 14C), thus inhibiting settlement and regrowth of the glioma cells (2nd column, Fig 14H). On the other hand, removal of the brain tissue on the left near BV (3rd column, Fig 14G) eliminated a large portion of invasive glioma cells but allowed the diffusion and active directed migration of glioma cells to the other hemisphere (Fig 14D), thus leading to recurrence of tumor cells in the right hemisphere (3rd column, Fig 14H). Elimination of the larger portion of brain tissue closer to the CC and BV inhibited local infiltration and regrowth in the other hemisphere (Fig 14E–14H). Thus an extensive resection of the glioma including CC and BVs may be effective in preventing invasiveness of tumor cells and recurrence of the tumor in the other side of hemisphere [8–10]. However, the extensive resection of the tumor mass including CC may cause side effects including cognitive impairment [52,53] despite its positively correlation of the longer survival rate [51]. Thus, if possible, a careful elimination of the primary tumor with better technologies for the right target preference may be necessary to gain optimal anti-cancer efficacy without causing the cognitive impairment.

**Fig 15:**
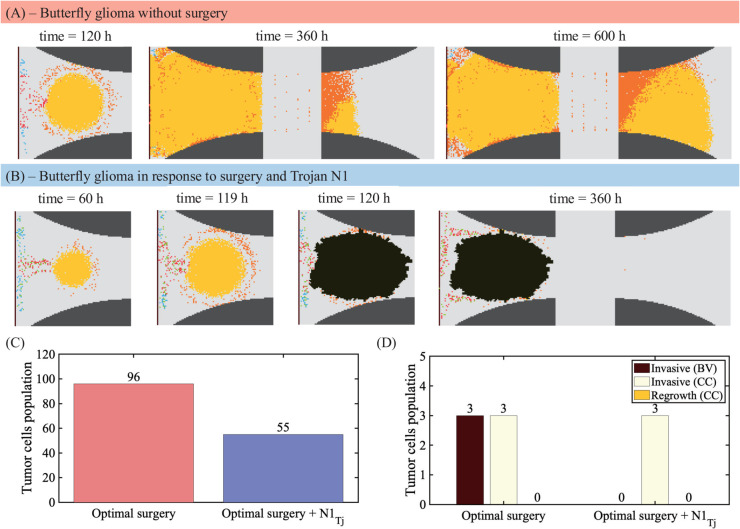
Anti-tumor efficacy of combination therapy (surgery and neutropphil-mediated agents) GBMs (A) Spatiotemporal dynamics of butterfly glioma without surgery. (B) Spatiotemporal dynamics of tumor cells in response to surgery at *t* = 120*h* and neutrophil-mediated anti-cancer agents (blue). (C) Tumor population at final time in response to optimal surgery and optimal surgery+N1 injection cases. (D) Population of infiltrative tumor cells along BV (brown), infiltrative tumor cells in the right hemisphere (white), and recurrent tumor cell population in the right hemisphere (comb lines) in optimal surgery and optimal surgery+N1 injection cases.

We finally investigated the additional effect of Trojan neutrophil injection in recurrence of GBM after aggressive surgery including BV and CC area (Fig 14). Without surgery, the invasive glioma cells located in the left hemisphere not only lead to fast growth but the invasive tumor cells actively crossed the CC and eventually form a large mass in the right hemisphere, generating butterfly glioma (Fig 15A). In addition to the massive elimination of tumor cells by surgical resection of the primary tumor at *t* = 120*h*, adjuvant injection of anti-cancer agents with neutrophils as a carrier (green dots in Fig 15B) resulted in extensive, additional killing of tumor cells in the BV area (Fig 15B, Fig 15C). During this process, the Trojan neutrophils strategy was also effective in killing escaping infiltrative tumor cells along BVs due to their source location (Fig 15D).

## Discussion

GBM is the most deadly form of brain cancer with a median overall survival of 14-17 months in spite of the conventional therapeutic approaches such as surgery, radiotherapy, and chemotherapy [98–101]. Despite their crucial role in cancer progression, the specific roles and fundamental mechanisms of neutrophils in the TME is poorly understood due to various challenges such as complex heterogeneity, short half-life, and technical difficulty in isolating and characterizing specific subsets [102]. While the classical anti-cancer roles of neutrophils imply a novel anti-tumor therapy [16,103,104], the activities of the N2 TANs and NETs are associated with tumor growth [26,105,106], angiogenesis [107–109], promotion of metastasis [110,111] via $\beta_{2}$-integrin [112] and cell cycle control [113]. An elevated NLR in blood was highly associated with poor clinical outcomes in GBM before treatment [114,115], after a surgery [116] and TMZ/RT therapies [117], thus recognizing NLR as an index for a poor prognostics [118]. In our model framework, we do not take into account the number of the whole lymphocytes. However, as the high level of NLR is a good indicator for high levels of N2 TANs in TME, we calculate the relative ratio of N2 TANs to N1 TANs, *SN21R* in Eq (12), in order to determine the relative level of activities of N2 TANs in given TME. In spite of lack of the exact scaling between NLR and SN21R, the high numbers of N2 TANs relative to the N1 TAN population can provide more accurate indicator to detect aggressive tumor growth. The transition between those two phenotypes of TANs is mediated by TGF-*β* and IFN-*β*, thus normalizing immune responses in the TME can increase anti-tumor efficacy [24–26].

The competition model between N1 and N2 TANs in our modeling framework allows us to get the transition between two phenotypes under various microenvironment conditions such as IFN-*β* and TGF-*β* (S2 Text). However, a large portion of N2 TANs reside in a typical TME where N1 TANs are either suppressed or converted to N2 TANs. However, injection of antibody against N2 TANs can transit N2 TANs back to N1 TANs in TME (S2 Text, Figs 5, 6, 7, 9, 11, and 13), which improves anti-tumor efficacy. As NLR can be a biomarker for brain cancer [16], in this study, we focus on the role of imbalance between N1 and N2 TANs in regulation of tumor growth, invasion and spread in the brain. Neutrophils infiltrate the glioma tissue in patients and the degree of infiltration is strongly associated with tumor grade [119]. Our study indicates that the prevalence of N2 TANs in the brain TME promotes tumor growth and spatial distribution of N1 and N2 TANs regulates patterns of growth and invasion of glioma cells via tumor-immune interaction and molecular feedback from TGF-*β* and IFN-*β*. This TAN-assisted tumor growth can be suppressed by a TGF-*β* inhibitor. Physical constraints from the distribution of white and gray matters in addition to this N2-to-N1 ratio in brain tissue also induce the nonlinear growth patterns.

Aggressive infiltration of tumor cells after surgical resection of the main tumor is the major cause of low survival rate of GBM patients due to tumor recurrence [120,121]. Thus, strategic control and eradication of these invisible invasive tumor cells in the brain TME is a major challenge in GBM treatment [41,42,97]. Inhibition of the pro-tumorigenic effect of N2 TANs on the periphery of resected tumor area after surgery can be offered as an adjuvant therapy in controlling remaining tumor cells. Xenotransplanted GBM cells showed a tendency to migrate along white matter tracts and CC [122]. In particular, CC can be used for fast tumor invasion, sometimes causing formation of a butterfly glioma [51], and tumor cells migrating through CC were elongated in shape with nuclear fragmentation from possible physical damage by shearing force [122]. (cf, see [123] for myosin II-mediated nuclear deformation in GBM cells through the narrow gap between normal cells). Poor prognosis [49,50] of gliomas infiltrating the corpus callosum led to debate on maximal safe resection relative to chemotherapy and radiation [124]. While maximal resection of the tumor mass near CC is positively correlated with longer overall survival [51], it often cause cognitive impairment [52,53]. When a tumor mass near CC is surgically removed, the remaining tumor cells spread with accelerated growth speed [46–48] and reach other hemisphere by crossing the CC.

Our work suggests that normalizing the immune responses and delivery of anti-cancer agents via Trojan neutrophils may be an effective way of eradicating glioma cells after surgery, reducing the chance of the critical infiltration through CC and formation of a larger mass, butterfly glioma. One of the key obstacle to improve clinical outcomes in GBM is to develop strategies of passing through BBB for direct delivery of drugs to the target [125]. In particular, BBB is still intact on the periphery of GBM where cancer cells aggressively invade normal brain tissue, restricting delivery of therapeutic agents to invasive margins and increasing the probability of tumor recurrence after treatment [126]. Various approaches are being developed to improve the delivery through BBB penetration in GBM using invasive and non-invasive approaches. Non-invasive form includes [125]:

- biological agents (peptides, transcytosis, oncolytic viruses, cell-mediated delivery, receptor-controlled BBB opening)
- intranasal delivery
- focused ultrasound (FUS-MB, MRgFUS)
- nanoscale delivery (liposomes, exosomes, nanomotors, nanoparticles)

These new approaches involve techniques such as hyperosmolar agents, junction-controlling molecules/particles to open tight junctions [125], blockers of efflux transporters, and receptor-regulated transcytosisi, and focused ultrasound with intravenous microbubbles [127,128]. However, the anti-tumor efficacy of the nanoparticle-based drug delivery systems after surgical resection of glioma is still poor due to fast decay of nanoparticles in circulation (a short half-life), systemic toxicity, and low accumulation in the intratumoral region [16]. Thus, cell-based drug delivery systems are suggested as an alternate, safe platform of drug delivery system for glioma [129–134]. Vectorization of drugs using endogenous cells has been considered for agent delivery to the brain [135–137]. In particular, neutrophils are good candidates for the delivery system due to (i) strong chemotactic movement toward the inflammatory sites, (ii) capacity of the pathogen elimination [138–140], (iii) their ability of crossing BBB/BTB and infiltration within the tumor mass [119,141–143], and (iv) TANs in the glioma TME [144] provide the constant recruitment of the neutrophils from blood [145]. In this work, while endogenous neutrophils provide either tumor-promoting or immune-suppressive roles in TME, the nanoparticle-carrying neutrophils increase the synergistic anti-tumor effect. A growing tumor on one side of hemisphere near CC can grow to a larger mass with a symmetric features, called butterfly glioma, leading to poor outcomes and serious cognitive impairment. In our work, these special Trojan horse neutrophils were injected to target the infiltrating glioma cells near CC after optimal surgical resection of the original tumor before local invasion, preventing inter-hemisphere spreading [8–10] and the critical recurrence in the other hemisphere. Surgery of GBM can induce local inflammation by secretion of inflammatory factors such as IL-8 [146,147] and TNF-a [148,149], leading to chemotactic movement of neutrophils toward the inflamed sector in the brain [138], thus, creating a good environment for the use of the Trojan neutrophils as a carrier. Since the surgical removal of the larger area of the tumor including CC increases the probability of serious side effects including cognitive impairment, the neutrophil-mediated delivery of nano anti-cancer particles can be a good alternative of rescuing the cancer-related cognitive changes [150].

While we did not take into account the dynamics of nanoparticles, the exact functions of BBB [125], ECM [97,151], and other important immune cells such as NK cells [19,20] and macrophages [126], and key signaling pathways such as STING [152] in this work, they play a critical role in regulating the tumor-immune cross-talk in the TME, designing the anti-cancer agents, and developing therapeutic strategies [125]. In particular, the detailed spatio-temporal dynamics of nanoparticles from Trojan N1 TANs can provide an optimally targeted anti-cancer strategy for both highly packed tumor core and infiltrative glioma cells in brain tissue. We will address these issues in future work.

## Supporting information

S1 Text Parameter Estimation. (PDF)

S2 Text Analysis of N1/N2 TANs model on spatially homogeneous system. (PDF)

S3 Text Glossary of selected terms and abbreviations. (PDF)

S4 Text Sensitivity Analysis of the basic unit (ODE model). (PDF)

S5 Text Sensitivity of key parameters to N1/N2 TANs dynamics and tumor growth (PDE model). (PDF)
