# Supplementary material for: How do tumor-associated neutrophils regulate the microenvironmental landscape of brain tumors: Delivery of nano-particles through BBB
Source: PLoS Comput Biol. 2026 Jan 23;22(1):e1013906. doi: 10.1371/journal.pcbi.1013906 (PMC12858081; doi:10.1371/journal.pcbi.1013906)
Supplement: S1 Text — (PDF) [file pcbi.1013906.s001.pdf]

Supporting Information

Haneol Cho, Junho Lee, Sean Lawler, and Yangjin Kim

S1: Parameter Estimation and Non-dimensionalization

1 Parameter Estimation

The parameter estimation for the N2/N1 TAN modules is done based on empirical observations reported in literature and mathematical assumptions to reflect a solid representation of tumor-immune communications. Key parameters were chosen to reflect growth rate of cells, spatial diffusivity constants, (random motion of cells and diffusion of molecules), decay rates of molecules and death/killing processes of cells, the mutual inhibition between N1 and N2 TANs, and transition dynamics. The process was also to ensure the essential, observed behaviors of the system, including positive feedback loops, mutual inhibition, and phenotypic transitions. The parameters in this work are estimated as follows:

μ_1, μ_2: The half-life of TANs within the circulation ($T_{1/2}$) was reported to be 17.3 hours [1]. By taking $T_{1/2} = 17.3\ h$, we get $\mu_1 = \mu_2 = \frac{ln(2)}{T_{1/2}} \sim 0.0401\ h^{-1}$.

μ_A: The half-life of an anti-Ly6G antibody, a N2 TANs antibody within the circulation ($T_{1/2}$), was reported to have a lower value which can be encompassed with a daily injection routine [2]. By taking $T_{1/2} = 24\ h$, we get $\mu_A = \frac{ln(2)}{T_{1/2}} \sim 0.0289\ h^{-1}$.

μ_S: The half-life of IFN_β within the circulation ($T_{1/2}$) was reported to be 5 hours [3]. By taking $T_{1/2} = 5\ h$, we get $\mu_S = \frac{ln(2)}{T_{1/2}} \sim 0.1386\ h^{-1}$.

μ_G: The half-life of TGF-β within the circulation ($T_{1/2}$) was reported to be 24 hours [4]. By taking $T_{1/2} = 24\ h$, we get $\mu_G = \frac{ln(2)}{T_{1/2}} \sim 0.028881\ h^{-1}$.

r: The basic growth rate of tumor cells was measured [5] and used in various experimental and theoretical studies [4,6]. We take $r = 8.40 \times 10^{-2}\ h^{-1}$.

D_1, D_2: The motility constant of TANs was measured and estimated to be in the range of $(1.1 \times 10^{-8} - 0)\ cm^2s^{-1}$ depending on various IL-8 concentrations $((5 \times 10^{-8} - 0)M)$ [7]. We take $D_1 = D_2 = 1.1 \times 10^{-9}\ cm^2s^{-1} = 3.96 \times 10^{-6}\ cm^2h^{-1}$.

D_A: The diffusion coefficient of N2 TANs antibody was measured and estimated to be $2.9 \times 10^{-7}\ cm^2s^{-1}$ [8]. We take $D_A = 1.044 \times 10^{-3}\ cm^2h^{-1}$.

D_S, D_G: The diffusion coefficient of TGF-β was measured and estimated to be 21.3 μm^2s^{-1} [9]. We take this value and assume that both TGF-β and IFN_β have the same diffusion coefficient, leading to $D_G = D_S = 7.668 \times 10^{-2}\ cm^2h^{-1}$.

D_n: The random motility constant of tumor cells was measured and used in various experimental and theoretical studies [4]. We take $D_n = 1.0 \times 10^{-9}\ cm^2s^{-1} = 3.6 \times 10^{-6}\ cm^2h^{-1}$.

Par	Description	Value	Refs
t^*	Time	1 h	
l	Length	1.0 cm	
N_1^*	Density of N1 TANs	1.0×10^6 $cells/cm^3$	[10, 11]
N_2^*	Density of N2 TANs	1.0×10^6 $cells/cm^3$	[10, 11]
n^*	Tumor density	1.0×10^9 $cells/cm^3$	[12–15]
G^*	TGF-β concentration	1 ng/cm^3	[16]
S^*	IFN-β concentration	10 ng/cm^3	[17]
A^*	N2 antibody	100 mg/cm^3	[18]

Table S1. Reference values in the model

2 Non-dimensionalization

Governing equations in a dimensional form are as follows:

$$\frac{\partial N_1}{\partial t} = \nabla\cdot(D_1(\mathbf{x})\nabla N_1) + r_1 N_1\left(1-\frac{N_1}{w_1 S + K_1}\right) - \alpha_1 N_1 N_2 - \mu_1 N_1, \tag{1}$$

$$\frac{\partial N_2}{\partial t} = \nabla\cdot(D_2(\mathbf{x})\nabla N_2) + r_2 N_2\left(1-\frac{N_2}{w_2 G + K_2}\right) - \alpha_2 N_2 N_1 - \mu_2 N_2 - \beta N_2 A, \tag{2}$$

$$\frac{\partial A}{\partial t} = \nabla\cdot(D_A(\mathbf{x})\nabla A) + \lambda_A I_{\Omega_A} - \mu_A A, \tag{3}$$

$$\frac{\partial n}{\partial t} = \nabla\cdot(D_n(\mathbf{x})\nabla n) + r\left(1 + r_N\frac{{N_2}^2}{k^2+{N_2}^2}\right)n\left(1-\frac{n}{n_0}\right) - \mu_n N_1 n, \tag{4}$$

$$\frac{\partial S}{\partial t} = \nabla\cdot(D_S(\mathbf{x})\nabla S) + \lambda_S I_S - \mu_S S, \tag{5}$$

$$\frac{\partial G}{\partial t} = \nabla\cdot(D_G(\mathbf{x})\nabla G) + \lambda_G n - \mu_G G. \tag{6}$$

For computational purposes we non-dimensionalize the variables and parameters in the mathematical models including those in Eqs (1)-(6) in the result section:

$$\begin{aligned}
&\bar{t} = \frac{t}{t^*},\ \overline{x} = \frac{x}{l},\ \overline{N}_1 = \frac{N_1}{N_1^*},\ \overline{N}_2 = \frac{N_2}{N_2^*},\ \overline{A} = \frac{A}{A^*},\ \overline{n} = \frac{n}{n^*},\ \overline{S} = \frac{S}{S^*},\ \overline{G} = \frac{G}{G^*},\ D_\dagger = \frac{l^2}{t^*},\\
&\overline{D}_1 = \frac{D_1}{D_\dagger},\ \overline{D}_2 = \frac{D_2}{D_\dagger},\ \overline{D}_A = \frac{D_A}{D_\dagger},\ \overline{D}_n = \frac{D_n}{D_\dagger},\ \overline{D}_S = \frac{D_S}{D_\dagger},\ \overline{D}_G = \frac{D_G}{D_\dagger},\ \overline{r}_1 = r_1 t^*,\\
&\overline{w}_1 = \frac{w_1 S^*}{N_1^*},\ \overline{K}_1 = \frac{K_1}{N_1^*},\ \overline{\alpha}_1 = \alpha_1 N_2^* t^*,\ \overline{r}_2 = r_2 t^*,\ \overline{w}_2 = \frac{w_2 G^*}{N_2^*},\ \overline{K}_2 = \frac{K_2}{N_2^*},\ \overline{\alpha}_2 = \alpha_2 N_1^* t^*,\\
&\overline{\beta} = \beta A^* t^*,\ \overline{\lambda}_A = \frac{\lambda_A t^*}{A^*},\ \overline{r} = r t^*,\ \overline{r}_N = r_N,\ \overline{k} = \frac{k}{N_2^*},\ \overline{n}_0 = \frac{n_0}{n^*},\ \overline{\lambda}_S = \frac{\lambda_S t^*}{S^*},\ \overline{\lambda}_G = \frac{\lambda_G n^* t^*}{G^*},\\
&\overline{\mu}_1 = \mu_1 t^*,\ \overline{\mu}_2 = \mu_2 t^*,\ \overline{\mu}_A = \mu_A t^*,\ \overline{\mu}_n = \mu_n N_1^* t^*,\ \overline{\mu}_S = \mu_S t^*,\ \overline{\mu}_G = \mu_G t^*.
\end{aligned} \tag{7}$$

In the simulation, we set $\overline{w}_1 = \overline{w}_2 = 1$.

After non-dlmensionalization of Eqns (1)-(6), we get governing equations in a dimensionless form as

follows:

$$\frac{\partial \overline{N}_1}{\partial \overline{t}} = \overline{\nabla} \cdot (\overline{D}_1(\mathbf{x})\overline{\nabla}\ \overline{N}_1) + \overline{r}_1\overline{N}_1\left(1 - \frac{\overline{N}_1}{\overline{w}_1\overline{S} + \overline{K}_1}\right) - \overline{\alpha}_1\overline{N}_1\overline{N}_2 - \overline{\mu}_1\overline{N}_1, \tag{8}$$

$$\frac{\partial \overline{N}_2}{\partial \overline{t}} = \overline{\nabla} \cdot (\overline{D}_2(\mathbf{x})\overline{\nabla}\ \overline{N}_2) + \overline{r}_2\overline{N}_2\left(1 - \frac{\overline{N}_2}{\overline{w}_2\overline{G} + \overline{K}_2}\right) - \overline{\alpha}_2\overline{N}_2\overline{N}_1 - \overline{\mu}_2\overline{N}_2 - \overline{\beta}\overline{N}_2\overline{A}, \tag{9}$$

$$\frac{\partial \overline{A}}{\partial \overline{t}} = \overline{\nabla} \cdot (\overline{D}_A(\mathbf{x})\overline{\nabla}\overline{A}) + \overline{\lambda}_A I_{\Omega_A} - \overline{\mu}_A\overline{A}, \tag{10}$$

$$\frac{\partial \overline{n}}{\partial \overline{t}} = \overline{\nabla} \cdot (\overline{D}_n(\mathbf{x})\overline{\nabla}\overline{n}) + \overline{r}\left(1 + \overline{r}_N\frac{\overline{N}_2^{\,2}}{\overline{k}^2 + \overline{N}_2^{\,2}}\right)\overline{n}\left(1 - \frac{\overline{n}}{\overline{n}_0}\right) - \overline{\mu}_n\overline{N}_1\overline{n}, \tag{11}$$

$$\frac{\partial \overline{S}}{\partial \overline{t}} = \overline{\nabla} \cdot (\overline{D}_S(\mathbf{x})\overline{\nabla}\overline{S}) + \overline{\lambda}_S I_{\overline{S}} - \overline{\mu}_S\overline{S}, \tag{12}$$

$$\frac{\partial \overline{G}}{\partial \overline{t}} = \overline{\nabla} \cdot (\overline{D}_G(\mathbf{x})\overline{\nabla}\overline{G}) + \overline{\lambda}_G\overline{n} - \overline{\mu}_G\overline{G}. \tag{13}$$

References

1. Steinbach KH, Schick P, Trepel F, Raffler H, Dohrmann J, Heilgeist G, et al. Estimation of kinetic parameters of neutrophilic, eosinophilic, and basophilic granulocytes in human blood. Blut. 1979;39:27–38.

2. Boivin G, Faget J, Ancey PB, Gkasti A, Mussard J, Engblom C, et al. Durable and controlled depletion of neutrophils in mice. Nat Commun. 2020;11(1):2762.

3. Salmon P, Cotonnec JYL, Galazka A, Abdul-Ahad A, Darragh A. Pharmacokinetics and pharmacodynamics of recombinant human interferon-beta in healthy male volunteers. J Interferon Cytokine Res. 1996;16(10):759–64.

4. Kim Y, Wallace J, Li F, Ostrowski M, Friedman A. Transformed Epithelial cells (TECs) and fibroblasts/myofibroblasts interaction in Breast Tumor: A Mathematical Model and Experiments. J Math Biol. 2009;in press:[DOI 10.1007/s00285–009–0307–2].

5. Catani JPP, Medrano RFV, Hunger A, Valle PD, Adjemian S, Zanatta DB, et al. Intratumoral Immunization by p19Arf and Interferon-beta Gene Transfer in a Heterotopic Mouse Model of Lung Carcinoma. Transl Oncol. 2016;9(6):565–574.

6. Kim Y, Lee D, Lee J, Lee S, Lawler S. Role of tumor-associated neutrophils in regulation of tumor growth in lung cancer development: A mathematical model. PLoS One. 2019;14(1):e0211041.

7. Moghe PV, Nelson RD, Tranquillo RT. Cytokine-stimulated chemotaxis of human neutrophils in a 3-D conjoined fibrin gel assay. J Immunol Methods. 1995;180(2):193–211.

8. Saltzman WM, Radomsky ML, Whaley KJ, Cone RA. Antibody Diffusion in Human Cervical Mucus. Biophys J. 1994;66(2 Pt 1):508–15.

9. Albro MB, Nims RJ, Cigan AD, Yeroushalmi KJ, Allistom T, Hung CT, et al. Accumulation of Exogenous Activated TGF-b in the Superficial Zone of Articular Cartilage. Biophys J. 2013;104(8):1794–804.

10. Wong WS, Lo AW, Siu LP, Leung JN, Tu SP, Tai SW, et al. Reference ranges for lymphocyte subsets among healthy Hong Kong Chinese adults by single-platform flow cytometry. Clin Vaccine Immunol. 2013;20(4):602–6.

11. Choi YW, Kim YH, Oh SY, Suh KW, Kim Y, Lee G, et al. Senescent Tumor Cells Build a Cytokine Shield in Colorectal Cancer. Advanced Science. 2021;8(4):2002497.

12. ODonoghue JA, Bardies M, Wheldon TE. Relationships between tumor size and curability for uniformly targeted therapy with beta-emitting radionuclides. J Nucl Med. 1995;36(10):1902–9.

13. Friedman A, Tian JP, Fulci G, Chiocca EA, Wang J. Glioma virotherapy: effects of innate immune suppression and increased viral replication capacity. Cancer Res. 2006;66(4):2314–9.

14. Kim Y, Yoo JY, Lee TJ, Liu J, Yu J, Caligiuri MA, et al. Complex role of NK cells in regulation of oncolytic virus-bortezomib therapy. Proc Natl Acad Sci USA. 2018;115(19):4927–4932.

15. Kim Y, Lee J, Lee D, Othmer HG. Synergistic effects of bortezomib-OV therapy and anti-invasive strategies in glioblastoma: a mathematical model. Cancers. 2019;11:215.

16. Serizawa M, Takahashi T, Yamamoto N, Koh Y. Combined treatment with erlotinib and a transforming growth factor-β type I receptor inhibitor effectively suppresses the enhanced motility of erlotinib-resistant non-small-cell lung cancer cells. J Thorac oncol. 2013;8(3):259–69.

17. Deng L, Liang H, Xu M, Yang X, Burnette B, Arina A, et al. STING-Dependent Cytosolic DNA Sensing Promotes Radiation-Induced Type I Interferon-Dependent Antitumor Immunity in Immunogenic Tumors. Immunity. 2014;41(5):843–52.

18. Haeri HH, Blaffert J, Schoffmann FA, Blech M, Hartl J, Garidel P, et al. Concentration Effects in the Interaction of Monoclonal Antibodies (mAbs) with their Immediate Environment Characterized by EPR Spectroscopy. Molecules. 2019;24:2528.
