# Supplementary material for: How do tumor-associated neutrophils regulate the microenvironmental landscape of brain tumors: Delivery of nano-particles through BBB
Source: PLoS Comput Biol. 2026 Jan 23;22(1):e1013906. doi: 10.1371/journal.pcbi.1013906 (PMC12858081; doi:10.1371/journal.pcbi.1013906)
Supplement: S2 Text — (PDF) [file pcbi.1013906.s002.pdf]

Supporting Information

Haneol Cho, Junho Lee, Sean Lawler, and Yangjin Kim

S2: Analysis of N1/N2 TANs model on spatially homogeneous system

We analyze the spatially homogeneous N1/N2 system. The governing equations of the spatially homogeneous system of the N1/N2 TAN populations in the main text in the presence of antibody are as follows:

$$\frac{dN_1}{dt} = r_1 N_1 \left(1 - \frac{N_1}{S_0 + K_1}\right) - \alpha_1 N_1 N_2 - \mu_1 N_1, \tag{1}$$

$$\frac{dN_2}{dt} = r_2 N_2 \left(1 - \frac{N_2}{G_0 + K_2}\right) - \alpha_2 N_2 N_1 - \mu_2 N_2 - \beta N_2 A, \tag{2}$$

$$\frac{dA}{dt} = \lambda_A - \mu_A A. \tag{3}$$

1 Stability analysis of N1/N2 TANs model

Preliminaries

By considering the steady states of the local N1/N2 system from (1)-(3)

$$0 = r_1 N_1^s \left(1 - \frac{N_1^s}{S_0 + K_1}\right) - \alpha_1 N_1^s N_2^s - \mu_1 N_1^s, \tag{4}$$

$$0 = r_2 N_2^s \left(1 - \frac{N_2^s}{G_0 + K_2}\right) - \alpha_2 N_2^s N_1^s - \mu_2 N_2^s - \beta N_2^s A^s, \tag{5}$$

$$0 = \lambda_A - \mu_A A^s, \tag{6}$$

and the obvious steady state of A variable, $A^s = \lambda_A/\mu_A$, we obtain N_1-nullclines and N_2-nullclines

$$N_1 \text{ nullcline} := \left\{ (N_1, N_2) | N_1 = 0 \text{ or } N_1 = \frac{S_0 + K_1}{r_1}(r_1 - \alpha_1 N_2 - \mu_1) \right\}, \tag{7}$$

$$N_2 \text{ nullcline} := \left\{ (N_1, N_2) | N_2 = 0 \text{ or } N_2 = \frac{G_0 + K_2}{r_2}(r_2 - \alpha_2 N_1 - \mu_2 - \beta A^*) \right\}. \tag{8}$$

Thus, we have the following set of steady state values in the $N_1 - N_2$ phase plane

$$(i) \quad \mathbf{S}_0 = (0, 0) \tag{9}$$

$$(ii) \quad \mathbf{S}_1 = \left((S_0 + K_1)\left(1 - \frac{\mu_1}{r_1}\right), 0 \right) \tag{10}$$

$$(iii) \quad \mathbf{S}_2 = \left(0, (G_0 + K_2)\left(1 - \frac{\mu_2 + \beta A^*}{r_2}\right) \right) \tag{11}$$

$$(iv) \quad \mathbf{S}_3 = (N_1^{**}, N_2^{**}) \tag{12}$$

where

$$N_1^{**} = \frac{\alpha_1(r_2 - \mu_2 - \beta A^*) - \dfrac{r_2}{G_0 + K_2}(r_1 - \mu_1)}{\alpha_1\alpha_2 - \dfrac{r_1 r_2}{(S_0 + K_1)(G_0 + K_2)}}, \tag{13}$$

$$N_2^{**} = \frac{\alpha_2(r_1 - \mu_1) - \dfrac{r_1}{S_0 + K_1}(r_2 - \mu_2 - \beta A^*)}{\alpha_1\alpha_2 - \dfrac{r_1 r_2}{(S_0 + K_1)(G_0 + K_2)}}. \tag{14}$$

Further restrictions on the positive steady states lead to a set of conditions as follows:

$$r_1 - \mu_1 > 0,\ r_2 - \mu_2 - \beta A^* > 0 \tag{15}$$

and either

$$\frac{r_1 - \mu_1}{\alpha_1} < \frac{(G_0 + K_2)(r_2 - \mu_2 - \beta A^*)}{r_2},\ \frac{r_2 - \mu_2 - \beta A^*}{\alpha_2} < \frac{(S_0 + K_1)(r_1 - \mu_1)}{r_1} \tag{16}$$

or

$$\frac{r_1 - \mu_1}{\alpha_1} > \frac{(G_0 + K_2)(r_2 - \mu_2 - \beta A^*)}{r_2},\ \frac{r_2 - \mu_2 - \beta A^*}{\alpha_2} > \frac{(S_0 + K_1)(r_1 - \mu_1)}{r_1}. \tag{17}$$

Stability analysis result and Calculation procedures

The stabilities of the each steady state $\mathbf{S}_i = (N_{1i}, N_{2i})$ $(i = 0, 2, 3)$ under two different inequality conditions Eq(16) and Eq(17) are summarized in the table as follows:

Table S1. Stabilities of steady states from the ODE model.

steady state	stability	
condition	when Eq (16) holds	when Eq (17) holds
$\mathbf{S}_0$	unstable	
$\mathbf{S}_1$	stable	saddle point
$\mathbf{S}_2$	stable	saddle point
$\mathbf{S}_3$	saddle point	stable

Considering this result and the parameter conditions from the previous part, we show how these stability results come from the given Jacobian matrix,

$$J(S_i) = \begin{pmatrix} r_1 - \dfrac{2r_1 N_{1i}}{S_0 + K_1} - \alpha_1 N_{2i} - \mu_1 & -\alpha_1 N_{1i} \\ -\alpha_2 N_{2i} & r_2 - \dfrac{2r_2 N_{2i}}{G_0 + K_2} - \alpha_2 N_{1i} - \mu_2 - \beta A^* \end{pmatrix}$$

for each steady state $S_i = (N_{1i}, N_{2i})$ $(i = 0, 1, 2, 3)$.

For the trivial steady state, $S_0 = (0, 0)$, the Jacobian matrix

$$J(S_0) = \begin{pmatrix} r_1 - \mu_1 & 0 \\ 0 & r_2 - \mu_2 - \beta A^* \end{pmatrix}. \tag{18}$$

Herein, eigenvalues of the given matrix are $\lambda_1 = r_1 - \mu_1, \lambda_2 = r_2 - \mu_2 - \beta A^*$ since $J(S_0)$ has a diagonal form, and from the condition (15), because all of the eigenvalues are positive, S_0 is an unstable steady state.

For the N1 dominant steady state, $S_1 = ((S_0 + K_1)(1 - \mu_1/r_1), 0)$, the Jacobian matrix

$$J(S_1) = \begin{pmatrix} -(r_1 - \mu_1) & -\alpha_1 \dfrac{S_0 + K_1}{r_1}(r_1 - \mu_1) \\ 0 & r_2 - \mu_2 - \beta A^* - \alpha_2 \dfrac{S_0 + K_1}{r_1}(r_1 - \mu_1) \end{pmatrix}. \tag{19}$$

The given Jacobian matrix is a triangular matrix, hence the eigenvalues are $\lambda_1 = -(r_1 - \mu_1)$, which has a negative sign from the condition (15), and $\lambda_2 = r_2 - \mu_2 - \beta A^* - \alpha_2(S_0 - K_1)(r_1 - \mu_1)/r_1$. The sign of the λ_2 is determined by whether the condition (16) or (17) holds: under the satisfaction of (16), λ_2 has a negative sign indicating S_1 be a stable steady state, while satisfying (17), λ_2 become positive which indicates that S_1 be a half-stable steady state, so-called a saddle point steady state.

Likewise, for the N2 dominant steady state, $S_2 = (0, (G_0 + K_2)(1 - (\mu_2 + \beta A^*)/r_2))$, the Jacobian matrix

$$J(S_2) = \begin{pmatrix} r_1 - \mu_1 - \alpha_1 \dfrac{G_0 + K_2}{r_2}(r_2 - \mu_2 - \beta A^*) & 0 \\ -\alpha_2 \dfrac{G_0 + K_2}{r_2}(r_2 - \mu_2 - \beta A^*) & -(r_2 - \mu_2 - \beta A^*) \end{pmatrix}. \tag{20}$$

Under the identical procedure as in the previous case $J(S_1)$, we can conclude that S_2 is a stable steady state when satisfying (16) and a half-stable steady state when satisfying (17).

Finally, the coexistence case, $S_3 = (N_1^{**}, N_2^{**})$ where nonnegative values of N_1^{**}, N_2^{**} are

$$N_1^{**} = \frac{\alpha_1(r_2 - \mu_2 - \beta A^*) - \dfrac{r_2}{G_0 + K_2}(r_1 - \mu_1)}{\alpha_1\alpha_2 - \dfrac{r_1 r_2}{(S_0 + K_1)(G_0 + K_2)}} \tag{21}$$

$$N_2^{**} = \frac{\alpha_2(r_1 - \mu_1) - \dfrac{r_1}{S_0 + K_1}(r_2 - \mu_2 - \beta A^*)}{\alpha_1\alpha_2 - \dfrac{r_1 r_2}{(S_0 + K_1)(G_0 + K_2)}}. \tag{22}$$

Because of the complicated structure included in this case, instead of directly calculating the eigenvalues of the $J(S_3)$, we evlauated the signs of the trace τ and determinant Δ to verify the stability of S_3 according to the conditions (16) and (17).

After proceeding algebras, we gained the trace of the given Jacobian matrix $J(S_3)$

$$\begin{aligned} \tau = \; & -\frac{1}{D}\left[\frac{r_1}{S_0 + K_1}\left(\alpha_1(r_2 - \mu_2 - \beta A^*) - \frac{r_2}{G_0 + K_2}(r_1 - \mu_1)\right)\right. \\ & \left. + \frac{r_2}{G_0 + K_2}\left(\alpha_2(r_1 - \mu_1) - \frac{r_1}{S_0 + K_1}(r_2 - \mu_2 - \beta A^*)\right)\right] \end{aligned} \tag{23}$$

where $D = \alpha_1\alpha_2 - r_1r_2/(S_0+K_1)(G_0+K_2)$.

Using the repersentaions from (21) and (22), we can rewrite it as

$$\tau = -\left(\frac{r_1}{S_0+K_1}N_1^{**} + \frac{r_2}{G_0+K_2}N_2^{**}\right).$$

Since both $N_1^{**}-$ and N_2^{**} have nonnegative values under the conditions (16) and (17), τ have nonpositive value. Hence, the S_3 is whether a stable steady state or a saddle point.

Meanwhile, the determinant of the $J(S_3)$ gainde from the several calculation processes

$$\Delta = (r_1 = \mu_1)(r_2 - \mu_2 - \beta A^*) - DN_1^{**}N_2^{**}.$$

Under the condition (17), $D < 0$ hence $\Delta > 0$, which concludes that S_3 is a stable steady state. However, when (17) holds, $D > 0$ and the sign of Δ varies according to the values between $(r_1 = \mu_1)(r_2 - \mu_2 - \beta A^*)$ and $DN_1^{**}N_2^{**}$.

2 Dynamics of N1/N2 TANs

Table S2. Stability of steady states in the N1/N2 TAN model.

	Stability			
Steady State	Mode I	Mode II	Mode III	Mode IV
$\mathbf{S}_0$	unstable			
$\mathbf{S}_1$	stable	unstable	saddle point	stable
$\mathbf{S}_2$	unstable	stable	saddle point	stable
$\mathbf{S}_3$			stable	saddle point
TME	Tumorigenic	Anti-tumorigenic	Co-existence (mixed)	Not relevant

We first investigate how the populations of N1 and N2 TANs are regulated in the local dynamics in response to two fixed stimuli from IFN-β ($S = S_0$), TGF-β ($G = G_0$) in the presence of the uniformly distributed N2 antibody, by considering the steady states of the local N1/N2 system from (1)-(3). Note that we set $\omega_1 = \omega_2 = 1$ in the nondimensionalization process in S1 Text. With the fixed point $A^s = \lambda_A/\mu_A$ for the antibody and N_1- and N_2-nullclines, we get four steady states $\mathbf{S}_0$, $\mathbf{S}_1$, $\mathbf{S}_3$, $\mathbf{S}_4$ in the (N_1, N_2) phase plane as in Eqs (9)-(12) in the previous section. Table S2 shows possible four modes (mode I, II, III, IV), based on the stability of the four different equilibrium points, that the dynamical system can obtain. We note that the obvious steady state $\mathbf{S}_0$ is always unstable. In mode I, $\mathbf{S}_1$ is stable and $\mathbf{S}_2$ is unstable, leading to N1-dominant condition, thus, anti-tumorigenic TME. On the other hand, in mode II, $\mathbf{S}_1$ is unstable and $\mathbf{S}_2$ is stable, leading to N2-dominant phase, tumorigenic TME. In mode III, both $\mathbf{S}_1$ and $\mathbf{S}_2$ are saddle points and $\mathbf{S}_3$ is a stable equilbirum in the middle of $N_1 - N_2$ phase plane, providing co-existence of N1 and N2 TANs in the system. In the case of single condition on parameters (Mode IV, Table S2), a persistent N1-dominant TME ($\mathbf{S}_1$; stable) and N2-dominant state ($\mathbf{S}_2$; stable) is possible in the absence of the co-existence of both TAN phenotypes ($\mathbf{S}_3$; saddle point). However, extinction of both N1 and N2 TANs is not possible as has been previously demonstrated experimentally [1–6]. Thus, in given biological system, the mode IV does not reflect the biological conditions in tumor microenvironment. Thus, from now on, we exclude mode IV and only consider the mode I, II, III.

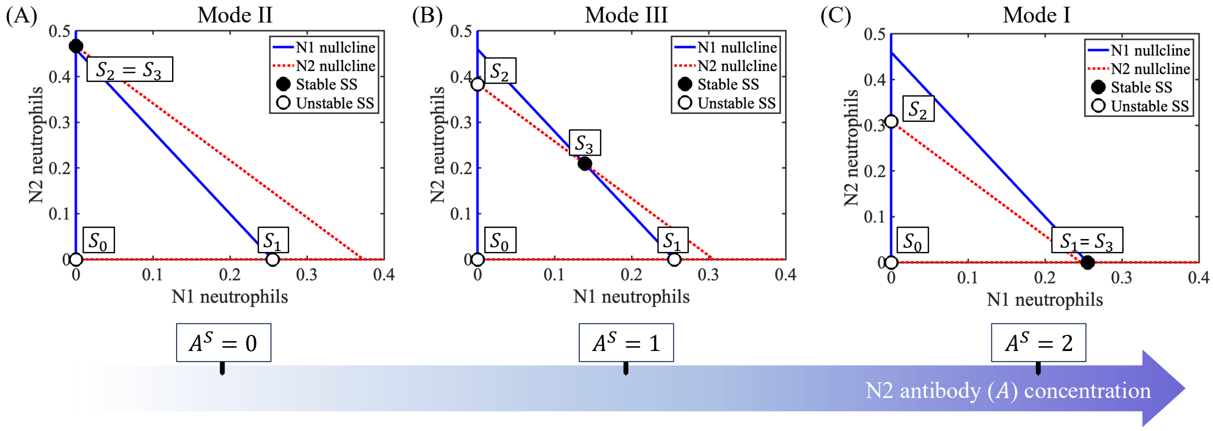

Figure S1. Nullclines of N1 and N2 TAN populations in the N_1-N_2 phase plane. (A-C) Nullclines (N1 (blue solid curves) and N2 (red dashed)) and stable (filled circle) and unstable (empty circles) steady states in the N1-N2 phase plane when $A^s = 0$ (A), $A^s = 1$ (B), $A^s = 2$ (C). While four possible steady states ($\mathbf{S}_0, \mathbf{S}_1, \mathbf{S}_2, \mathbf{S}_3$) are possible, key controller, N2 antibody (A^s) can induce the phenotypic transition from the N2-dominant phase ($\mathbf{S}_2$ in (A)) to co-existence of those N1/N2 TANs ($\mathbf{S}_3$ in (B)), and to the N1-dominant state ($\mathbf{S}_1$ in (C)). The equilibrium point at $\mathbf{S}_0$ is always unstable under conditions in the model, excluding unrealistic oncologic conditions as in experiments [1–6]. Parameters: $\alpha_1 = 0.5, \alpha_2 = 1.5,\ K_1 = 0.3,\ K_2 = 0.5, \mu_1 = 4.01 \times 10^{-2},\ r_1 = 0.27,\ \mu_2 = 4.01 \times 10^{-2},\ r_2 = 0.6,\ \beta = 0.1,\ S_0 \equiv 0,\ G_0 \equiv 0$.

Fig S1 illustrates how the fixed biochemical conditions from antibody input in a TME can determine an extinction of both or one of TANs as well as co-existence of N1 and N2 TANs. In a typical TME condition, the immune system choose the N2-dominant status, *i.e.,* stable $\mathbf{S}_2$ condition where N1 TANs are suppressed and N2 TANs are promoted (Fig S1A). Presence of low levels of antibody ($A^s = 1$) leads to Mode III in Table S2 where the system induces the co-existence of N1 and N2 TANs ($\mathbf{S}_3$, stable) with unstable (saddle points) status at $\mathbf{S}_1$ and $\mathbf{S}_2$ equilibria (Fig S1B). In the presence of a high level of antibody ($A^s = 2$), $\mathbf{S}_3$ converges to the stable $\mathbf{S}_2$, leading to a typical immune-active status where N1 TANs persist and N2 TANs are suppressed (Fig S1C). Thus, as the level of antibody (A^s) is perturbed from mode III, the system nullifies the co-existence state ($\mathbf{S}_3$) by shifting it to either $\mathbf{S}_1$ or $\mathbf{S}_2$ with stability changes at $\mathbf{S}_1$ and $\mathbf{S}_2$ (mode I or mode II in Table S2). For example, as the N2 antibody concentration (A^s) increases from zero ($A^s = 0$), the stable steady state of TANs transits from the N2 type ($\mathbf{S}_2$ in Fig S1A; $A^s = 0$) to the mixed N1/N2 type ($\mathbf{S}_3$ in Fig S1B; $A^s = 1$) and to the N1 type ($\mathbf{S}_1$ in Fig S1C; $A^s = 2$):

Mode II	$\rightarrow$	Mode III	$\rightarrow$	Mode I
(Tumorigenic, N2+N1-)		(Mixed, N2+N1+)		(Anit-tumorigenic, N2-N1+)

This indicates that suppression of N2 TANs in TME by injection of antibodies can transit the N2 type to N1 type, leading to higher anti-tumor efficacy.

Fig S2(A-C) shows the trajectories of solutions of N1/N2 TANs in response to various injection rates of N2 antibody ($\lambda_A = 0, 0.0289, 0.289$). As the antibody injection rate is increased ($\lambda_A = 0 \rightarrow 0.0289 \rightarrow 0.289$), the antibody level ($A$) is increased, resulting in the transition of the unique steady state of N1/N2 TANs from N_2 status (Fig S2A) to co-existence status (Fig S2B) and to N_1 mode (Fig S2C). Fig S2(D-E) shows the time courses of the N1 TAN population (Fig S2D) and N2 TAN population (Fig S2E) for corresponding cases. Thus, as λ_A is increased, the tumor size is decreased (Fig S2F) due to the enhanced

anti-tumor efficacy from N1 TANs (Fig S2D) and decreased N2 TAN populations (Fig S2E), illustrating overall anti-tumor efficacy of N2 antibody.

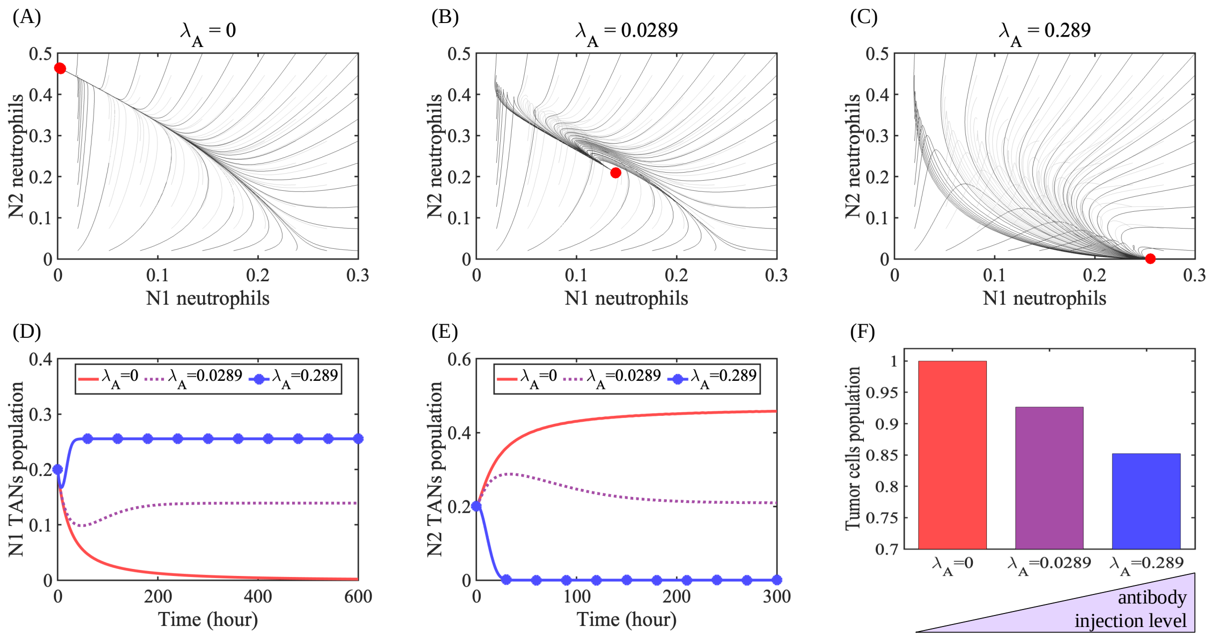

Figure S2. Effect of N2 antibody in the TAN dynamics on tumor growth. (A-C) Trajectories of solutions $(N_1(t), N_2(t))$ in the N1-N2 phase plane when $\lambda_A = 0$ (A), $\lambda_A = 0.0289$ (B), and $\lambda_A = 0.289$ (C). All solutions converge to the unique stable steady state marked with a red circle. (D-E) Time course of the N1 (D) and N2 (E) TAN population for the corresponding λ_A's. (F) Tumor cell populations at final time for $\lambda_A = 0, 0.0289, 0.289$. Parameter: same as in Fig S1.

A relative immune balance between IFN-β and TGF-β can change the landscape of N1 and N2 TAN composition in TME, which can determine tumor growth [1]. IFN-β up-regulates N1 TANs (dashed, Fig S3A) and suppresses N2 TANs (dashed, Fig S3B), resulting in slower tumor growth (dashed, Fig S3C). On the other hand, TGF-β increases the N2 TANs (empty circle, Fig S3B) and decreases the N1 TAN population (empty circle, Fig S3A), which eventually promotes tumor growth (empty circle, Fig S3C) relative control (blue solid line, Fig S3C). These model predictions are consistent with experimental observations on the pro-tumorigenic effect of N2 TANs and anti-tumor effect of N1 TANs in the TME [1–6]. These indicate that the relative balance of IFN-β (S) and TGF-β (G) and increase/decrease in these molecules as well as biophysical conditions such as white matter and gray matter will shape the solution flow to infer N1 or N2-dominent TANs in TME as well as co-existence of N1/N2 TANs.

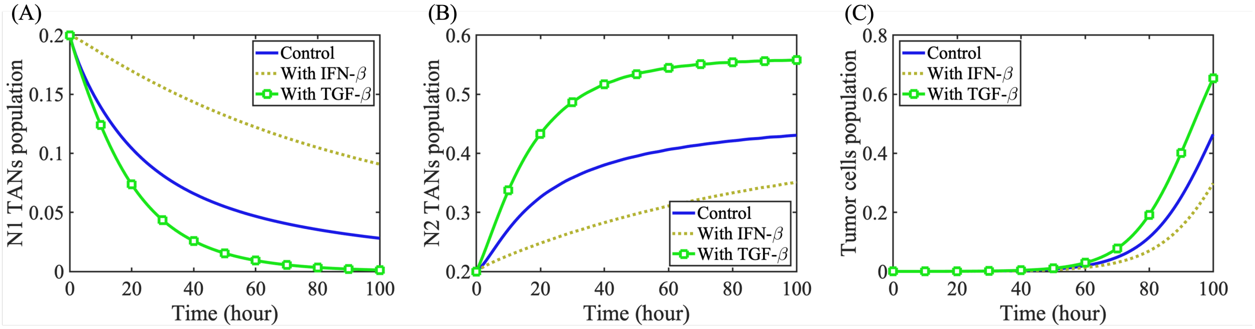

Figure S3. Effect of IFN-β, and TGF-β on the local dynamics and transition between N1 and N2 TAN, and tumor growth. (A-C) Time courses of populations of N1 TANs (A), N2 TANs (B), and tumor cells (C) in the presence of IFN-β (yellow dotted line) and TGF-β (green line with circles) ($S \equiv 0.1$, $G \equiv 0.1$) relative to control ($S \equiv 0 \equiv G$). Initial conditions: $N_1(0) = 0.2$, $N_2(0) = 0.2$.

References

1. Shaul ME, Levy L, Sun J, Mishalian I, Singhal S, Kapoor V, et al. Tumor-associated neutrophils display a distinct N1 profile following TGFbeta modulation: A transcriptomics analysis of pro- vs. antitumor TANs. Oncoimmunology. 2016;5(11).

2. Hagerling C, Werb Z. Neutrophils: Critical components in experimental animal models of cancer. Semin Immunol. 2016;28(2):197–204.

3. Sionov RV, Fridlender ZG, Granot Z. The multifaceted roles neutrophils play in the tumor microenvironment. Cancer Microenviron. 2015;8(3):125–58.

4. Swierczak A, Mouchemore KA, Hamilton JA, Anderson RL. Neutrophils: important contributors to tumor progression and metastasis. Cancer Metast Rev. 2015;34:735–751.

5. Liang W, Ferrara N. The complex role of neutrophils in tumor angiogenesis and metastasis. Cancer Immunol Res. 2016;4:83–91.

6. Fridlender ZG, Sun J, Kim S, Kapoor V, Cheng G, Ling L, et al. Polarization of tumor-associated neutrophil phenotype by TGF-beta: N1 versus N2 TAN. Cancer Cell. 2009;16(3):183 – 194. doi:10.1016/j.ccr.2009.06.017.
