# Supplementary material for: How do tumor-associated neutrophils regulate the microenvironmental landscape of brain tumors: Delivery of nano-particles through BBB
Source: PLoS Comput Biol. 2026 Jan 23;22(1):e1013906. doi: 10.1371/journal.pcbi.1013906 (PMC12858081; doi:10.1371/journal.pcbi.1013906)
Supplement: S3 Text — (PDF) [file pcbi.1013906.s003.pdf]

Supporting Information

Haneol Cho, Junho Lee, Sean Lawler, and Yangjin Kim

S3: Glossary of selected terms and abbreviations

- *Angiogenesis*: Development of new blood vessels, especially in tissues where circulation has been impaired by trauma or disease, e.g., cancer.
- *Apoptosis*: Programmed cell death characterized by nuclear breakdown and removal of remains by phagocytes.
- *BBB*: Blood–brain barrier.
- *BED*: Biologically effective dose.
- *BTB*: Blood-tumor barrier.
- *CC*: Corpus callosum. A bundle of nerve fibers that allow the brain's left and right hemispheres to communicate.
- *Cytokine*: Extracellular signaling protein that acts as a local mediator in cellecell communication. Those involved in taxis are sometimes called chemokines.
- *ECM*: Extracellular matrix.
- *Chemotaxis*: Movement of a motile cell or organism in a direction corresponding to a gradient of increasing concentration of a particular substance called 'chemoattractant'.
- *GBM*: Glioblastoma multiforme.
- *Growth factor*: an extracellular signaling molecule that stimu- lates a cell to grow or proliferate, e.g., vascular endothelial growth factor (VEGF) and fibroblast growth factor (FGF).
- *IFN-β*: Interferon beta.
- *Metastasis*: the process by which cancer spreads from the site of initiation of the primary tumor to distant locations in the body. This occurs via either the circulatory system or the lymphatic system.
- *MRI*: Magnetic Resonance Imaging.
- *NETs*: Neutrophil extracellular traps.
- *NK cells*: Natural killer cells.
- *NLR*: Neutrophil to lymphocyte ratio.
- *ODE*: Ordinary differential equation.
- *PDE*: Partial differential equation.
- *TAMs*: Tumor-associated macrophages.
- *TANs*: Tumor-associated neutrophils.
- *TGF-β*: Transforming growth factor-beta.
- *TME*: Tumor microenvironment.
