# Supplementary material for: How do tumor-associated neutrophils regulate the microenvironmental landscape of brain tumors: Delivery of nano-particles through BBB
Source: PLoS Comput Biol. 2026 Jan 23;22(1):e1013906. doi: 10.1371/journal.pcbi.1013906 (PMC12858081; doi:10.1371/journal.pcbi.1013906)
Supplement: S4 Text — (PDF) [file pcbi.1013906.s004.pdf]

Supporting Information

Haneol Cho, Junho Lee, Sean Lawler, and Yangjin Kim

S4: Sensitivity Analysis of the basic unit (ODE model)

Sensitivity analysis

In order to investigate the sensitivity of some parameters in the model, we performed a sensitivity analysis on twenty parameters to examine correlations with key variables. Based on the sensitivity analysis suggested by Marino et al. [1] and MATLAB files (http://malthus.micro.med.umich.edu/lab/usadata/), we used general Latin hypercube sampling (LHS) scheme and partial rank correlation coefficient (PRCC) for twenty parameters $(r_1, r_2, K_1, K_2, \alpha_1, \alpha_2, \mu_1, \mu_2, \beta, \lambda_A, \mu_A, \lambda_S, \mu_S, \lambda_G, \mu_G, r, r_N, k, n_0, \mu_n)$ in the model for all model variables (N_1, N_2, A, S, G, n).

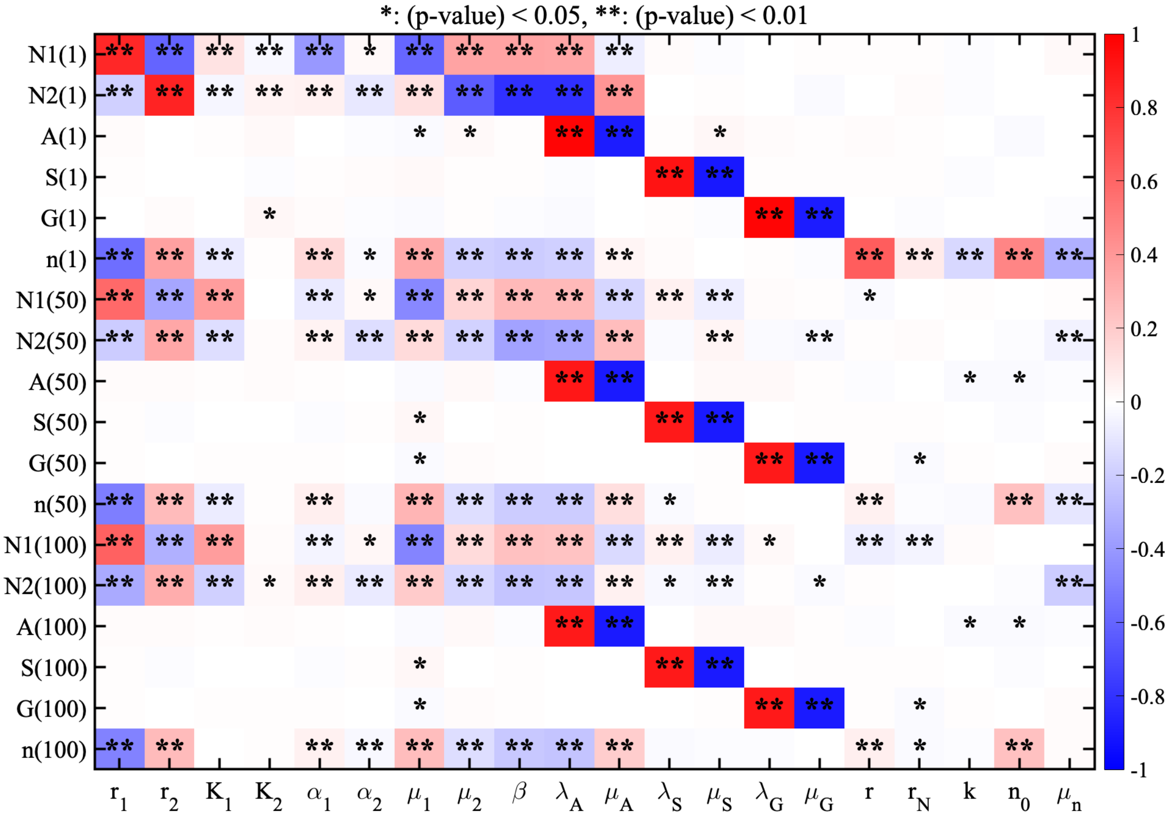

Figure S1. Sensitivity analysis. LHS scheme and PRCC are used to analyze the mathematical model without diffusions at time $t = 1, 50, 100$ hour with a sample size 10,000 [1]. Each colored box indicates the PRCC value of each variable for parameters in the model: red (or blue) represents the positive (or negative) correlation.

We have chosen a range of each parameter and divided them with 10,000 uniform-length subintervals, and calculated PRCC values and p-values at $t = 1, 50, 100$ hour. Each PRCC value lies on the interval $[-1, 1]$ with a sign indicating the positive (or negative) correlation.

Sensitivity analysis results are summarized in Fig S1. Color of each cell indicates either positive (red) or negative (blue) correlation of the parameter to given variable. The star sign in each cell denotes p-value associated with the PRCC value: a single star for $(p-value) < 0.05$ and double star for $(p-value) < 0.01$. Based on these statistical analysis results, we conclude the following: (i) The parameters associated with proliferation of N1 TANs (r_1, K_1) and suppression of N2 TANs $(\mu_2, \beta, \lambda_A)$ are positively correlated with the N1 TAN population while inhibition parameters of N1 TANs (α_1, μ_1) and promotion rates of N2 TANs (r_2, K_2, μ_A) have a negative correlation with the N1 TAN population. Especially, r_1 (or μ_1) has a very strong positive (or negative) correlation with the N1 TANs population. (ii) The parameters r_2, μ_A have a positive correlation with N2 TANs but μ_2, β, λ_A have a negative correlation with the N2 population. (iii) The tumor cell population shares a similar set of parameters with N2 TANs for positive and negative correlations, except n_0. The carrying capacity n_0 of tumor cells is positively correlated with tumor population as expected. (iv) The source term (or decay rate) for each cytokines (N2 antibody (A), IFN$_\beta$ (S), and TGF-β (G)) has a strong positive correlation with the main corresponding variable. For example, the TGF-β source (λ_G) is positively correlated with the TGF-β concentration and the decay rate of IFN$_\beta$ (μ_S) is negatively correlated with the IFN$_\beta$ level at given time points.

References

1. Marino S, Hogue IB, Ray CJ, Kirschner DE. A methodology for performing global uncertainty and sensitivity analysis in systems biology. Journal of Theoretical Biology. 2008;254(1):178–196.
