# Supplementary material for: How do tumor-associated neutrophils regulate the microenvironmental landscape of brain tumors: Delivery of nano-particles through BBB
Source: PLoS Comput Biol. 2026 Jan 23;22(1):e1013906. doi: 10.1371/journal.pcbi.1013906 (PMC12858081; doi:10.1371/journal.pcbi.1013906)
Supplement: S5 Text — (PDF) [file pcbi.1013906.s005.pdf]

Supporting Information

Haneol Cho, Junho Lee, Sean Lawler, and Yangjin Kim

S5: Sensitivity of key parameters to N1/N2 TANs dynamics and tumor growth (PDE model)

Fig S1 shows effect of key parameters ($r_1, r_2, \alpha_1, \alpha_2$, λ_S, λ_G) on dynamics of the N1/N2 TANs and tumor growth. The population of N1 TANs (or N2 TANs) is strongly affected by r_1 (or r_2) as they determine the key growth component, and these changes lead to changes in the tumor size by suppressing or promoting tumor growth (Fig S1(A-B)). The competition between N1 and N2 TANs (α_1, α_2) also changes the dynamics of their counterpart in the spectrum of TANs, which affects the tumor size (Fig S1(C-D)). On the other hand, perturbations in secretion rates of IFN$_\beta$ and TGF-β affect N1 and N2 TANs, respectively, but their influence on the tumor growth is indirect relative to N1/N2 competition and direct growth rates, due to various TME conditions such as diffusion and relative positions of the tumor (Fig S1(E-F)).

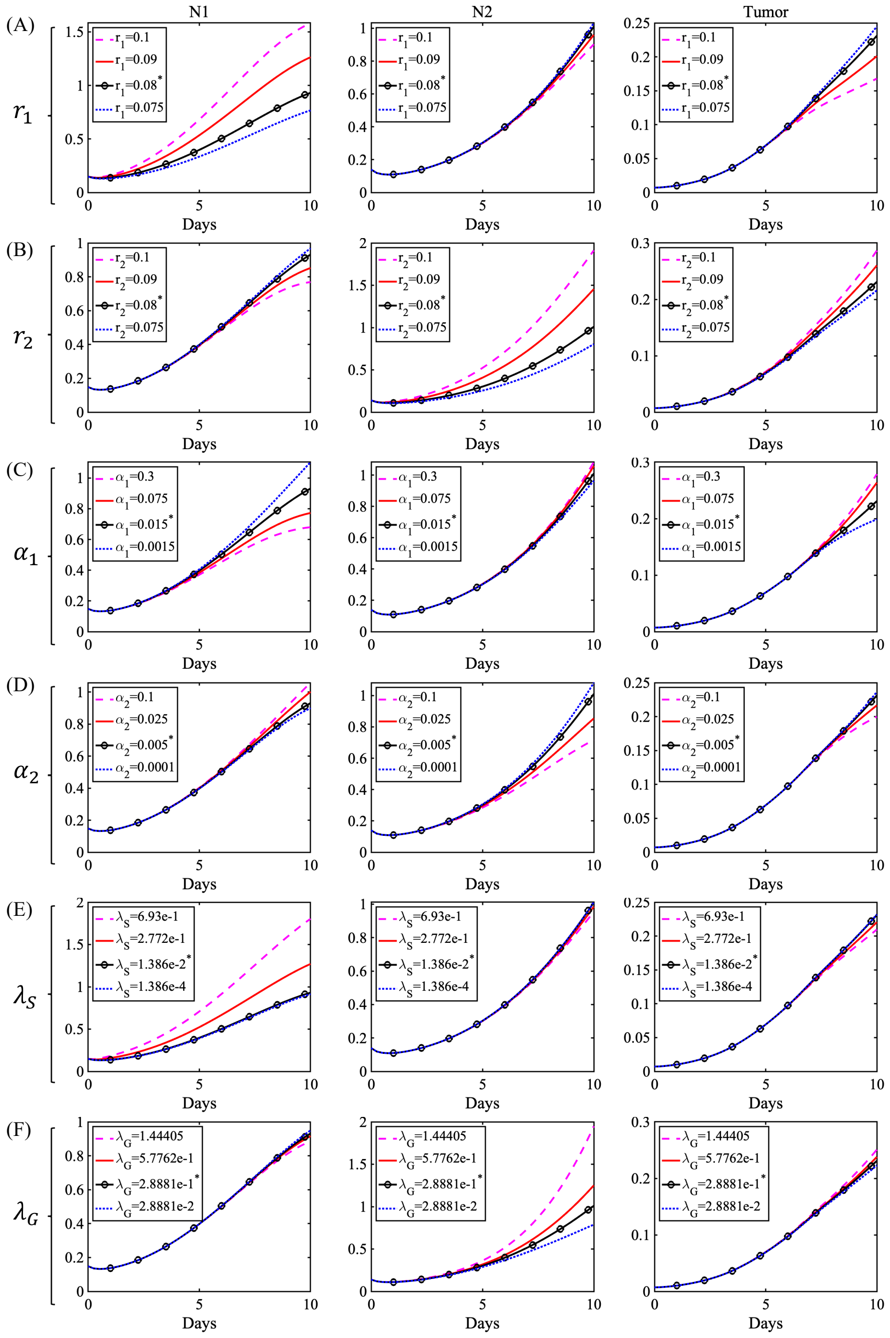

Figure S1. Effect of key parameters on dynamics of the N1/N2 TANs and tumor growth Time courses of populations of N1 TANs, N2 TANs, and tumor cells when the key parameter (r_1 (A), r_2 (B), α_1 (C), α_2 (D), λ_S (E), λ_G (F)) varies from the base value (marked in asterisk (*)).
